# Binary or Nonbinary Fission? Reproductive Mode of a Predatory Bacterium Depends on Prey Size

**DOI:** 10.1128/mbio.00772-23

**Published:** 2023-05-10

**Authors:** Karolina Pląskowska, Łukasz Makowski, Agnieszka Strzałka, Jolanta Zakrzewska-Czerwińska

**Affiliations:** a Department of Molecular Microbiology, Faculty of Biotechnology, University of Wrocław, Wrocław, Poland; Max-Planck-Institut fur terrestrische Mikrobiologie

**Keywords:** *Bdellovibrio bacteriovorus*, FtsZ, cell cycle, chromosome replication, predator

## Abstract

Most bacteria, including model organisms such as Escherichia coli, Bacillus subtilis, and Caulobacter crescentus, reproduce by binary fission. However, some bacteria belonging to various lineages, including antibiotic-producing *Streptomyces* and predatory *Bdellovibrio,* proliferate by nonbinary fission, wherein three or more chromosome copies are synthesized and the resulting multinucleoid filamentous cell subdivides into progeny cells. Here, we demonstrate for the first time that the predatory bacterium Bdellovibrio bacteriovorus reproduces through both binary and nonbinary fission inside different prey bacteria. Switching between the two modes correlates with the prey size. In relatively small prey cells, B. bacteriovorus undergoes binary fission; the FtsZ ring assembles in the midcell, and the mother cell splits into two daughter cells. In larger prey cells, B. bacteriovorus switches to nonbinary fission and creates multiple asynchronously assembled FtsZ rings to produce three or more daughter cells. Completion of bacterial cell cycle critically depends on precise spatiotemporal coordination of chromosome replication with other cell cycle events, including cell division. We show that B. bacteriovorus always initiates chromosome replication at the invasive pole of the cell, but the spatiotemporal choreography of subsequent steps depends on the fission mode and/or the number of progeny cells. In nonbinary dividing filaments producing five or more progeny cells, the last round(s) of replication may also be initiated at the noninvasive pole. Altogether, we find that B. bacteriovorus reproduces through bimodal fission and that extracellular factors, such as the prey size, can shape replication choreography, providing new insights about bacterial life cycles.

## INTRODUCTION

Most well-studied model bacteria, such as Escherichia coli, Bacillus subtilis, and Caulobacter crescentus, reproduce by binary fission (1). In these bacteria, the newly synthesized sister chromosomes are segregated into two nascent cells prior to the completion of cell division. However, some bacteria belonging to various lineages, including antibiotic-producing *Streptomyces* and predatory *Bdellovibrio*, proliferate by nonbinary fission (2–4). In such cases, more than two chromosome copies are synthesized, and the resulting multinucleoid filamentous cell subdivides into single-nucleoid progeny cells (5). Thus, in growing nonbinary bacteria, DNA replication is not directly followed by cell division.

While the Dynamics of chromosome replication in relation to multipoint and synchronous septation have been relatively well studied in *Streptomyces* spp. (6–8), these aspects remain unexplored in *Bdellovibrio*. The most widespread species of this predatory genus is Bdellovibrio bacteriovorus, which preys on other Gram-negative bacteria. A growing body of research indicates that B. bacteriovorus could be used as a natural antibiotic agent (called a “living antibiotic”) in both health care and agriculture. B. bacteriovorus has been shown to kill various pathogens (e.g., Klebsiella pneumoniae, *Salmonella*, and Vibrio parahaemolyticus) in different animal models (rats, mice, chicks, and shrimps) (9–12).

In recent years, B. bacteriovorus has received considerable research attention, not only because of its potential application as a “living antibiotic,” but also because of its intriguing life cycle, which is reminiscent of that of a bacteriophage. The life cycle of B. bacteriovorus consists of two phases as follows: a free-living nonreplicative attack phase and an intracellular, nonbinary reproductive phase (13–15). The free-living bacterium is asymmetric, with a flagellum and a pilus (i.e., an invasive pole) located at the opposite poles of cell. In the free-living phase, B. bacteriovorus uses its flagellum to move at speeds of up to 160 μm/s in search of prey (16). After engaging in pilus-mediated attachment to the prey’s outer membrane, B. bacteriovorus employs various glycosidases and peptidases to pierce the outer membrane and peptidoglycan and then enters the periplasmic space (17, 18). At the beginning of the reproductive phase, the prey cell dies and bloats into a spherical structure called a prey bdelloplast, inside of which the predatory cell “consumes” the prey contents (i.e., it degrades the prey’s macromolecules and reuses them for its own growth) and proliferates by nonbinary fission (3, 4). During this phase, the single chromosome is copied multiple times. Chromosome replication is initiated near the invasive pole (3, 19) and, after a few rounds of chromosome replication, the resulting multinucleoid filamentous cell divides into various (even or odd) numbers of progeny cells. Usually, three to six B. bacteriovorus progeny cells are formed when E. coli serves as the prey (4). The flagellated progeny cells are released into the environment to repeat the cycle.

Our current knowledge of the B. bacteriovorus cell cycle is largely based on studies employing E. coli as a model prey. In order to understand how B. bacteriovorus can be applied to combat bacterial infections caused by pathogens, studies on its life cycle in different Gram-negative pathogenic bacteria are of paramount importance.

In the present study, we provide key insights into the mode and dynamics by which B. bacteriovorus proliferates in different pathogens, Proteus mirabilis, Salmonella enterica, and Shigella flexneri, that are on the World Health Organization (WHO) global priority pathogen list of antibiotic-resistant bacteria (20). Currently, at least 700,000 people worldwide die each year due to drug-resistant infections, and according to WHO predictions, this number could rise to 10 million by 2050 (21). Using a set of B. bacteriovorus fluorescent reporter strains, we performed real-time observation of the major steps of the predator’s cell cycle in different pathogens. These steps include chromosome replication and cell division (septation). We reveal that the chromosome replication choreography and division mode differ across the analyzed prey bacteria and demonstrate, for the first time, that the predatory bacterium, B. bacteriovorus, undergoes binary or nonbinary fission depending on the size of its prey cell.

## RESULTS

### Proliferation of B. bacteriovorus in different pathogens.

To investigate the influence of the prey cell on B. bacteriovorus proliferation, we chose the following three different preys that are on the WHO global priority pathogen list of antibiotic-resistant bacteria (20): Proteus mirabilis, Salmonella enterica, and Shigella flexneri (for details see Table S1 in the supplemental material). Firstly, we assessed the ability of B. bacteriovorus to prey on the different pathogens by generating predatory killing curves using the pathogens as preys (see Fig. S1A in the supplemental material). In this analysis, a decrease in prey cell optical density reflected the lysis of cells by B. bacteriovorus. Interestingly, the shapes of the curves are different among the analyzed prey bacteria. The fastest decrease in prey cell optical density was observed for S. flexneri cells. Similar dynamics of predation was observed for S. enterica. While the dynamics of B. bacteriovorus predation on the third tested pathogen P. mirabilis cells was substantially different: there was a slow decrease in optical density at the beginning of measurements followed by a rapid decrease in optical density (Fig. S1A).

10.1128/mbio.00772-23.2 TABLE S1 Bacterial species, strains, plasmids, and primers used in this study. Download Table S1, DOCX file, 0.02 MB. Copyright © 2023 Pląskowska et al. 2023 Pląskowska et al. https://creativecommons.org/licenses/by/4.0/ This content is distributed under the terms of the Creative Commons Attribution 4.0 International license.

10.1128/mbio.00772-23.4 FIG S1 Basic features of B. bacteriovorus proliferation in different preys. (A) Kill curves of B. bacteriovorus during predation on three different preys as follows: P. mirabilis, S. enterica, and S. flexneri. Solid lines show the model fitted using a four-parameter Weibull function. (B) Correlation between the prey cell length and the diameter of the bdelloplast formed after B. bacteriovorus enters the prey (*n* = 100 for each prey). Download FIG S1, TIF file, 0.4 MB. Copyright © 2023 Pląskowska et al. 2023 Pląskowska et al. https://creativecommons.org/licenses/by/4.0/ This content is distributed under the terms of the Creative Commons Attribution 4.0 International license.

**FIG 1:**
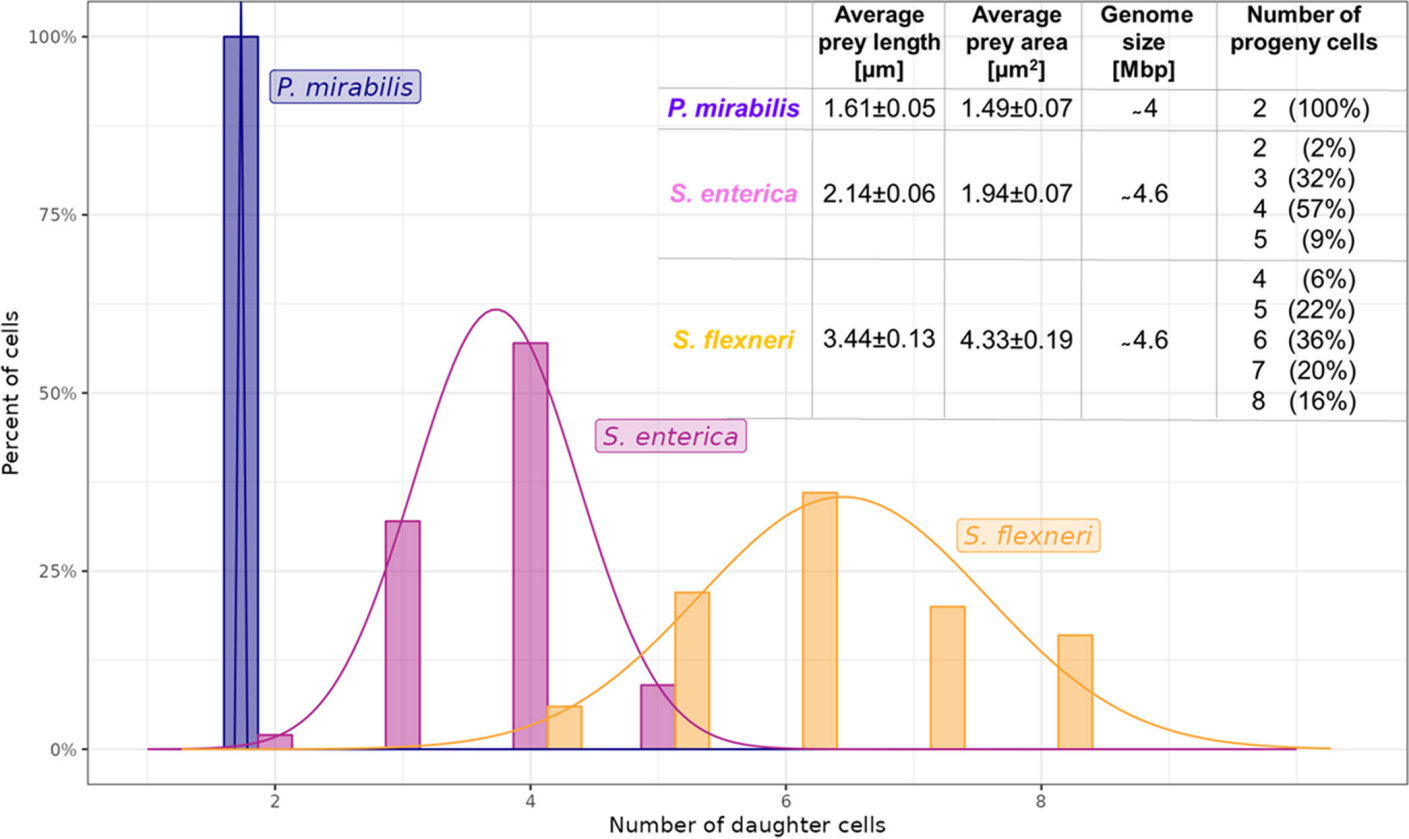
Number of progeny cells of B. bacteriovorus formed in different preys (*n* = 100 for each prey).

To further elucidate the differences in predation dynamics (mirrored by the killing curves), we measured different parameters of the prey and predator cells, including the prey cell size before infection, the diameter of the formed bdelloplasts (Fig. S1B), and the number of predatory cells released after prey cell lysis (Fig. 1) using single cell microscopic data. The average size of the prey cell varied and was reflected by differences in the bdelloplast diameter (Fig. S1B). As expected, the diameter of the bdelloplast was positively correlated with the length (and the area) of the prey cell (calculated Pearson correlation coefficient, *R* = 0.87). The number of predatory cells formed inside the bdelloplast depends on the prey cell size (Fig. 1). In contrast to E. coli and other larger prey cells, predation on a tiny P. mirabilis cell resulted in the generation of two progeny cells (100%, *n* = 100 prey cells analyzed) (Fig. 1). More progeny cells were formed in larger-sized prey cells as follows: two to five (usually 3 or 4) and four to eight (usually 5 to 7) progeny cells were produced from S. enterica and S. flexneri, respectively (Fig. 1). To prove that the positive correlation between number of predator offspring and prey size is independent of the bacterial species, we analyzed E. coli S17-1 cells of different sizes (see Table S2 in the supplemental material). Our measurements confirmed that the number of predator offspring scales with the E. coli size (a similar relationship was also observed for S. flexneri as a prey) (Table S2). Taken together, the results indicate that the infection dynamics of B. bacteriovorus predation is related to the number of progeny cells released from a single prey cell. The predatory killing curves illustrated this correlation (Fig. S1A); at the beginning of infection, when more progeny predators were released (e.g., from S. flexneri cells), more prey cells were killed as reflected by a faster decrease in the prey cell optical density (Fig. S1A).

10.1128/mbio.00772-23.3 TABLE S2 Number of B. bacteriovorus progeny cells formed in E. coli S17-1 and S. flexneri preys that differ in sizes. Download Table S2, DOCX file, 0.01 MB. Copyright © 2023 Pląskowska et al. 2023 Pląskowska et al. https://creativecommons.org/licenses/by/4.0/ This content is distributed under the terms of the Creative Commons Attribution 4.0 International license.

In summary, our results suggest for the first time that B. bacteriovorus divides not only by nonbinary (more than two, even or odd daughter cells) but also by binary (two daughter cells in P. mirabilis) fission, and the mode of division appears to depend on the prey size.

### Life cycle of B. bacteriovorus in different pathogens.

As we observed prey-dependent differences in predation dynamics, we next decided to closely investigate the main stages of the B. bacteriovorus reproductive phase (i.e., chromosome replication, segregation, and filament septation) in P. mirabilis, S. enterica, and S. flexneri at the single-cell level using time-lapse fluorescence microscopy (TLFM). For this purpose, we constructed a set of B. bacteriovorus reporter strains that allowed us to monitor the localization of DnaN, ParB, and FtsZ proteins involved in the chromosome replication, segregation, and filament septation, respectively (for details, see Table 1). All B. bacteriovorus reporter strains exhibited a killing curve for E. coli (see Fig. S2A in the supplemental material) similar to that of the wild-type strain. Production of the fusion proteins was confirmed using Western blotting (Fig. S2B).

**TABLE 1:** Characteristic features of the B. bacteriovorus fluorescent reporter strains used in this study

Strain	Task/characteristic features
DnaN-mNeonGreen	Replisome(s) localization
	DnaN, the replisome β sliding clamp subunit responsible for DNA polymerase III processivity
mNeonGreen-ParB/DnaN-mCherry	Simultaneous localization of segrosome(s) and replisome(s)
	ParB, the segrosome protein binding *parS* sequence(s) that are localized near the *oriC* region
FtsZ-mNeonGreen/DnaN-mCherry	Simultaneous localization of divisiome(s) and replisome(s)
	FtsZ, the divisiome protein assembling into a Z-ring at the future site of cell division

10.1128/mbio.00772-23.5 FIG S2 Characteristics of B. bacteriovorus strains used in this study. (A) Kill curves of B. bacteriovorus strains. Predation of B. bacteriovorus strains DnaN-mNeonGreen, mNeonGreen-ParB/DnaN-mCherry, FtsZ-mNeonGreen/DnaN-mCherry, and wild-type on E. coli ML35. Obtained kill curves are similar, which indicates that genetic manipulations in B. bacteriovorus strains did not affect the effectiveness of lysis of the prey. (B) Western blots of whole-cell protein extracts from B. bacteriovorus were probed with mNeonGreen antibody to confirm proper protein production. Line 1, mNeonGreen-ParB (64.4 kDa); line 2, DnaN-mNeonGreen (68.2 kDa); line 3, FtsZ-mNeonGreen (85.4 kDa); line 4, DnaN-mCherry (69.2 kDa); WT, negative control, B. bacteriovorus HD100 wild-type. One star reflected detected proteins with fusion and double star reflected fluorescent protein mNeonGreen (26.6 kDa) or mCherry (26.7 kDa). Download FIG S2, TIF file, 0.3 MB. Copyright © 2023 Pląskowska et al. 2023 Pląskowska et al. https://creativecommons.org/licenses/by/4.0/ This content is distributed under the terms of the Creative Commons Attribution 4.0 International license.

### Chromosome replication and segregation.

**FIG 2:**
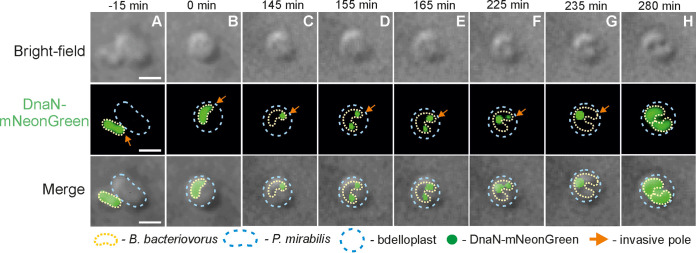
Spatiotemporal analysis of chromosome replication in a B. bacteriovorus cell growing in P. mirabilis. Time-lapse analysis of a representative B. bacteriovorus cell showing the localization of replisome(s) (green) in a predatory cell growing inside the P. mirabilis bdelloplast. (A) Attachment of B. bacteriovorus to a P. mirabilis cell. (B) Bdelloplast formation; time = 0 min. (C) Appearance of the first DnaN-mNeonGreen focus at the invasive pole of a B. bacteriovorus cell, indicating initiation of chromosome replication. (D to G) Further steps of chromosome replication, including splitting of replication forks (the appearance of the second DnaN-mNeonGreen focus at the former flagellar pole) and merging of replication forks at the midcell. (H) Termination of chromosome replication (disassembly of replisomes) and formation of two progeny cells. Photos represent merged bright-field and green fluorescence images. Scale bar = 1 μm. The full time-lapse is shown in Video S1 at https://figshare.com/authors/Jolanta_Zakrzewska-Czerwinska/14854612.

Chromosome replication and segregation dynamics of B. bacteriovorus were studied in P. mirabilis, being the simplest model as only two daughter cells are produced (Fig. 1). Firstly, we intended to observe the localization of the replisome(s) during the reproductive phase. We therefore replace the native DnaN of B. bacteriovorus with a DnaN-mNeonGreen. The appearance and disappearance of DnaN-mNeonGreen foci indicate the assembly and disassembly, respectively, of replisome complexes and thus correspond to the initiation and termination of DNA replication (22). DNA synthesis started (i.e., a DnaN-mNeonGreen focus appeared) consistently at the invasive pole (3, 19) in bdelloplasts (*n* = 100, 96%) (Fig. 2C; see also Video S1 at https://figshare.com/authors/Jolanta_Zakrzewska-Czerwinska/14854612) at 46 ± 4 min after bdelloplast formation (Table 2). The second focus appeared at 31 ± 4 min (*n* = 100) after the first initiation of DNA replication and subsequently migrated to the opposite pole (the pole that contained the flagellum before prey attack, hereinafter called the “former flagellar pole”). The pole-to-pole distance was covered in 11 ± 2 min (*n* = 100). Next, the replisomes relocated from the cell poles to a midcell position (Fig. 2; see also Video S1 at the URL mentioned above). Both replisomes remained at this position until the end of chromosome replication, which was indicated by diffusion of the DnaN-mNeonGreen fluorescent foci. It is worth noting that we never observed more than two foci during the reproductive phase in P. mirabilis-derivative bdelloplasts (*n* = 100). Thus, the DnaN-mNeonGreen fluorescent foci presumably represent the two replication forks that, during DNA synthesis, transiently split at the invasive pole and then merge in the midcell position. The average duration of chromosome replication was 144 ± 7 min (*n* = 100).

**TABLE 2:** Comparison of the average appearance times of replisome(s) and segrosome(s) in B. bacteriovorus cells during proliferation in different preys

Prey	Avg time^*a*^ (min)							
	1st DnaN focus	2nd ParB focus	Migration of new *oriC* to the opposite pole	2nd DnaN focus	3rd ParB focus	3rd DnaN focus	4th ParB focus	Duration of life cycle
P. mirabilis	46 ± 4	69 ± 4	19 ± 2	79 ± 6				252 ± 7
S. enterica	46 ± 5	65 ± 5	18 ± 2	119 ± 7	138 ± 8	163 ± 9	182 ± 10	288 ± 7
S. flexneri ^*b*^	44 ± 5	76 ± 6	17 ± 2	128 ± 7	144 ± 7	191 ± 9	211 ± 8	379 ± 11

a Average times were obtained after statistical analysis performed on 100 predator filaments for each prey. For all measured values, 95% confidence interval was calculated, assuming a normal distribution of results. The appearance of fluorescent foci was measured from the bdelloplast formation (time zero). The time of newly synthesized *oriC* (i.e., ParB focus) migration to the opposite pole was measured from the duplication of ParB focus.

b In B. bacteriovorus filament growing inside S. flexneri, more than three DnaN and four ParB foci were observed, but data are not presented.

**FIG 3:**
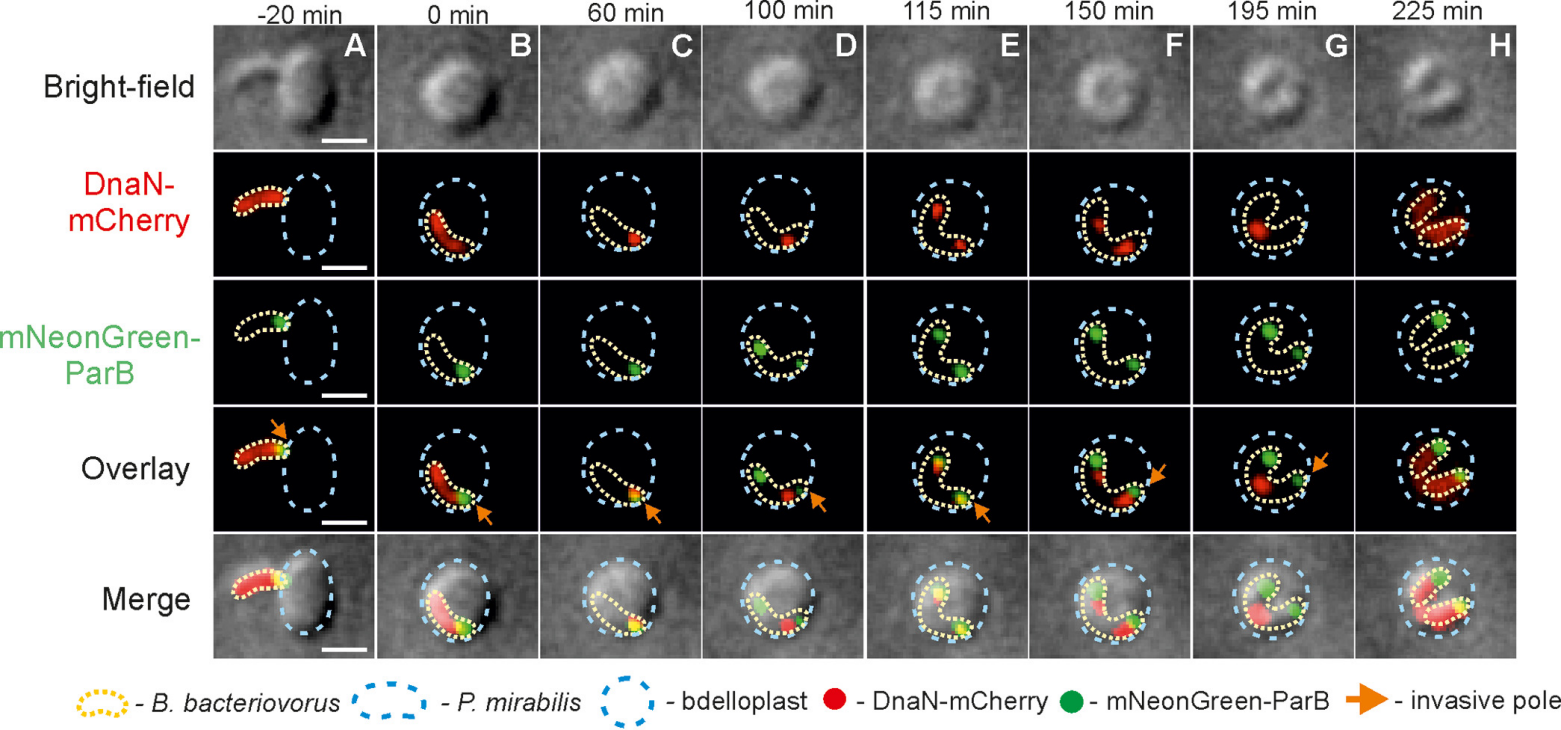
Time-lapse analysis of chromosome replication and *oriC* segregation in a B. bacteriovorus cell growing in P. mirabilis. Localization of replisome(s) (red) and *oriC* (i.e., ParB complex) (green) in a predatory B. bacteriovorus cell growing inside a P. mirabilis bdelloplast. (A) Attachment of B. bacteriovorus to a P. mirabilis cell. (B) Bdelloplast formation; time = 0 min. (C) Appearance of the first DnaN-mCherry focus at the invasive pole of a B. bacteriovorus cell, indicating initiation of chromosome replication. (D to G) Duplication of the *oriC* region (i.e., mNeonGreen-ParB focus) at the invasive pole; thereafter, one of the two *oriC* regions migrates to the opposite pole and remains there until the end of the cell cycle. Further steps of chromosome replication include splitting of replication forks (the appearance of the second DnaN-mCherry focus at the former flagellar pole) and merging of replication forks at the midcell. (H) Termination of chromosome replication (disassembly of replisomes) and formation of two progeny cells. Photos represent merged bright-field and fluorescence (red and green) images. Scale bar = 1 μm. The full time-lapse is shown in Video S2 at https://figshare.com/authors/Jolanta_Zakrzewska-Czerwinska/14854612.

**FIG 4:**
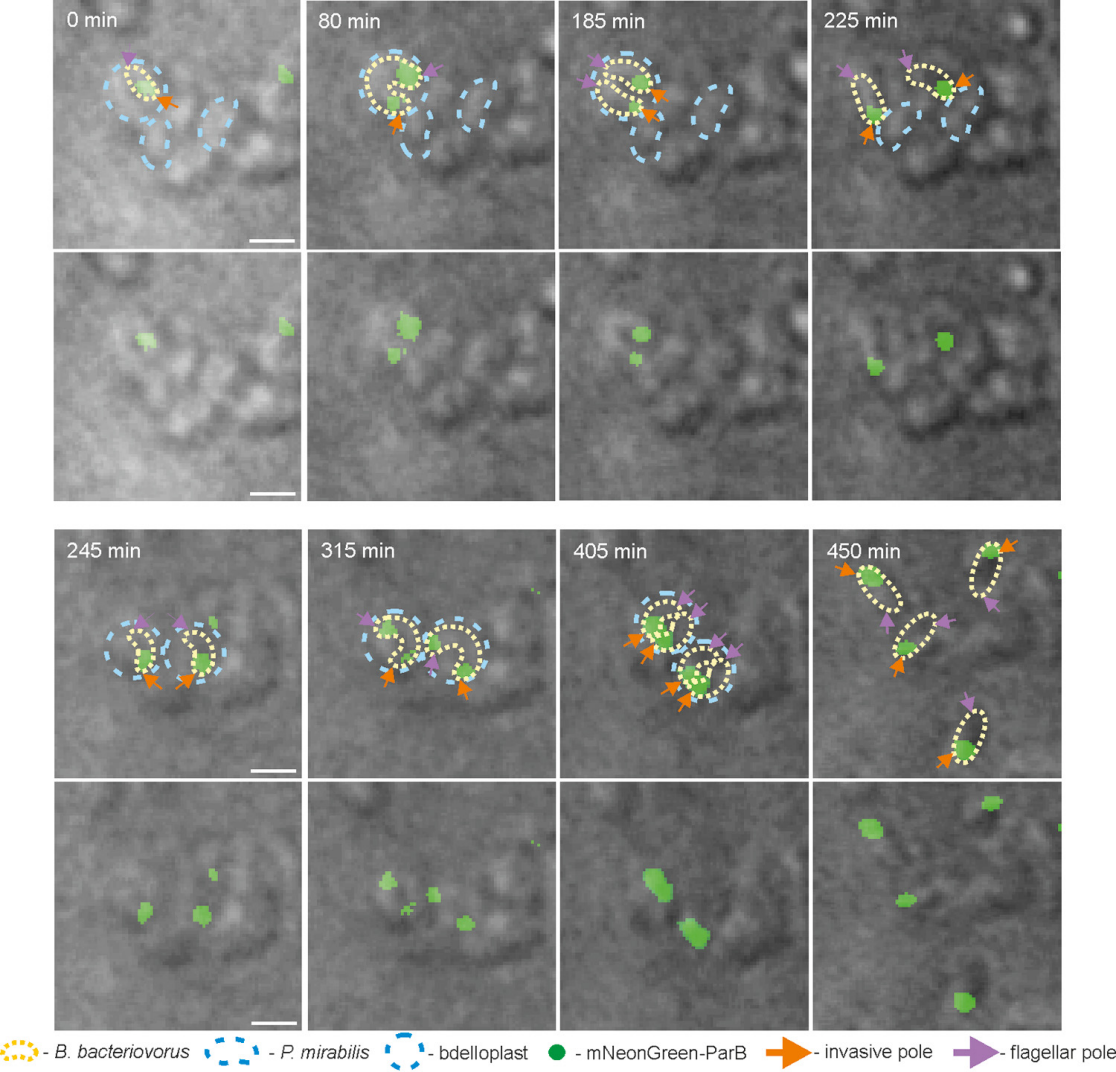
Conversion of the flagellated pole of the mother cell into an invasive pole inherited by a daughter cell during B. bacteriovorus proliferation in P. mirabilis. Time-lapse microscopy of B. bacteriovorus growing inside P. mirabilis shows that the flagellated pole of the mother cell (violet arrow) becomes an invasive pole (orange arrow) that is inherited by the daughter cell. The newly formed invasive pole has a visible ParB complex (i.e., *oriC* region) and is functional since the daughter cell attacks the next P. mirabilis cell through that pole. Photos represent merged bright-field images. The B. bacteriovorus cell and the bdelloplast are marked by yellow and blue dotted lines, respectively. Scale bar = 1 μm. The full time-lapse is shown in Video S3 at https://figshare.com/authors/Jolanta_Zakrzewska-Czerwinska/14854612.

We next investigated the dynamics of chromosome segregation in relation to DNA replication for B. bacteriovorus growing in P. mirabilis. For this purpose, we created allelic replacements of ParB and DnaN with fluorescent fusion proteins in B. bacteriovorus (see Table 1; see also Table S1), which allowed us to simultaneously monitor both processes in a single cell. The ParB focus (i.e., *oriC* region) was observed as previously described (3, 23) at the invasive pole (see Fig. 3A). Notably, B. bacteriovorus mNeonGreen-ParB/DnaN-mCherry strain exhibited a proliferation-phase fluorescent pattern for DnaN-mCherry (Fig. 3; see also Video S2 at https://figshare.com/authors/Jolanta_Zakrzewska-Czerwinska/14854612) consistent with that observed in the B. bacteriovorus DnaN-mNeonGreen strain (Fig. 2; see also Video S1 at the URL mentioned above). In this system, DNA replication was followed by chromosome segregation (Fig. 3; see also Video S2 at the URL mentioned above). Shortly after the initiation of DNA synthesis (at 16 ± 3 min and 69 ± 4 min after bdelloplast formation) (see Table 2), one of the newly replicated *oriC* regions visible as a ParB focus (ParB complexes colocalize with the *oriC* region) (3) started to migrate to the opposite cell pole, reaching its destination after 19 ± 2 min (Table 2). In contrast to replisomes, the segrosomes did not relocate to midcell but instead remained at the filament poles (Fig. 3; see also Video S2 at the URL mentioned above). Careful examination of dozens of proliferating B. bacteriovorus cells in P. mirabilis (*n* = 45; as an example, see Fig. 4; see also Video S3 at the URL mentioned above) using an agarose pad in combination with ibidi cell-imaging dishes (predatory cells are less motile in this system) (19) revealed that the former flagellar pole (i.e., the one opposite to the invasive pole marked by a distinct ParB complex) (3, 23) during the attack phase later became an invasive pole with a visible ParB complex. Thus, the two poles arising after division become the flagellar poles. The newly created invasive pole is fully functional as the daughter cell attacks the next prey cell through that pole (Fig. 4; see also Video S3 at the URL mentioned above).

**FIG 5:**
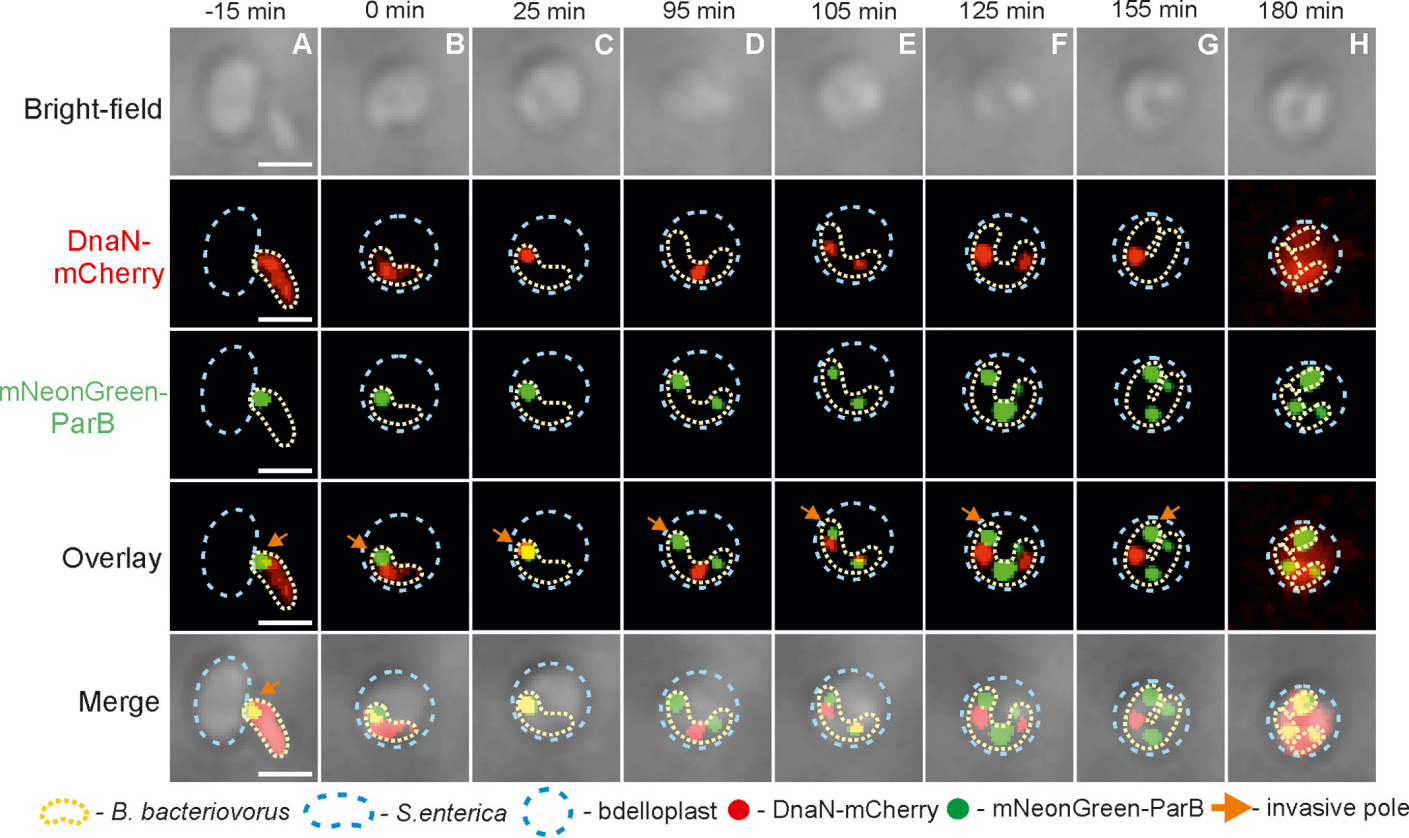
Dynamics of chromosome replication and *oriC* segregation in a B. bacteriovorus cell growing in S. enterica: formation of three daughter cells. (A) Attachment of B. bacteriovorus to a S. enterica cell. (B) Bdelloplast formation; time = 0 min. (C) Appearance of the first DnaN-mCherry focus at the invasive pole of a B. bacteriovorus cell, indicating initiation of chromosome replication. (D) Duplication of the *oriC* region (i.e., mNeonGreen-ParB focus) at the invasive pole; thereafter, one of the two *oriC* regions migrates to the opposite pole and one of the two DnaN-mCherry foci follows behind the newly replicated *oriC* region that is already attached to the opposite pole. (E and F) Reinitiation of DNA replication from the mother chromosome located at the invasive pole, migration of both replisomes toward the opposite pole, and the appearance of the third ParB focus at the middle of the filament. (G) Disappearance of the DnaN-mCherry focus located near the former flagellar pole, indicating termination of the first-round DNA replication. (H) Termination of the second round of chromosome replication and formation of three daughter cells. Photos represent merged bright-field and fluorescence (red and green) images. The B. bacteriovorus cell and the bdelloplast are marked by yellow and blue dotted lines, respectively. Scale bar = 1 μm. The full time-lapse is shown in Video S4 at https://figshare.com/authors/Jolanta_Zakrzewska-Czerwinska/14854612.

**FIG 6:**
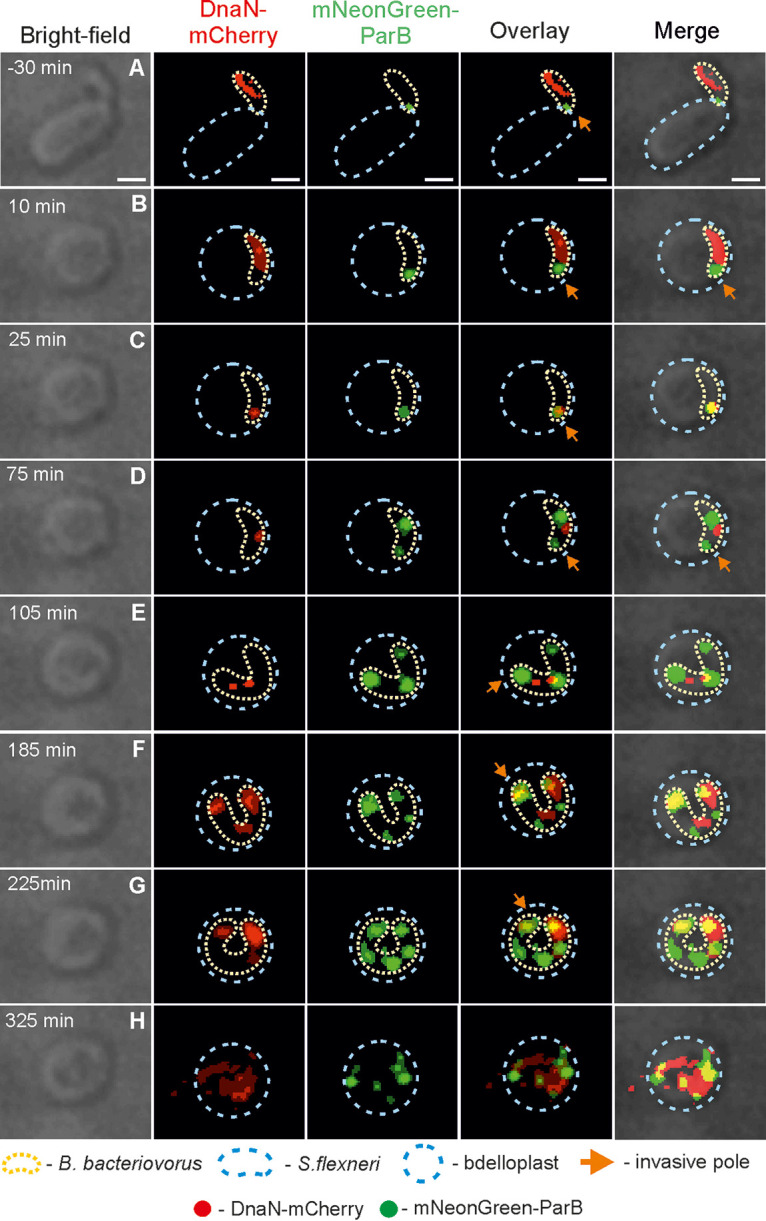
Dynamics of chromosome replication and *oriC* segregation in a B. bacteriovorus cell during proliferation in S. flexneri. (A) Attachment of B. bacteriovorus to an S. flexneri cell. (B) Bdelloplast formation; time = 0 min. (C) Appearance of the first DnaN-mCherry focus at the invasive pole of a B. bacteriovorus cell, indicating initiation of chromosome replication. (D and E) Replisome follows the newly replicated *oriC* region, indicating the progress of DNA replication. The appearance of a second DnaN-mCherry focus (reinitiation of chromosome replication), movement of the two replisomes toward opposite poles, and development of the third mNeonGreen-ParB focus in the middle of the filament. (F and G) Reinitiation of DNA replication from the chromosome located at the former flagellar pole. Appearance of another ParB signals reflects the emergence of a newly replicated *oriC* region. (H) Termination of DNA replication. Photos represent merged bright-field and fluorescence (red and green) images. The B. bacteriovorus cell and the bdelloplast are marked by yellow and blue dotted lines, respectively. Scale bar = 1 μm. The full time-lapse is shown in Video S6 at https://figshare.com/authors/Jolanta_Zakrzewska-Czerwinska/14854612.

To understand how chromosome replication and segregation dynamics are affected by the production of multiple progeny cells, we fed B. bacteriovorus on larger than P. mirabilis cells, S. enterica (usually 3 or 4 daughter cells) and S. flexneri (4 to 8 daughter cells) (differences in sizes among analyzed preys are shown in Fig. 1 and Fig. S1B). As expected, in these preys, chromosome replication was also initiated at the invasive cell pole of B. bacteriovorus (Fig. 5 and 6; see also Fig. S3 in the supplemental material; additionally see Videos S4 to S6 at https://figshare.com/authors/Jolanta_Zakrzewska-Czerwinska/14854612) at 46 ± 5 min after bdelloplast formation in S. enterica and at 44 ± 5 min in S. flexneri consistent with previous experiments (Table 2). In S. enterica and S. flexneri, one of the newly replicated B. bacteriovorus *oriC* regions (i.e., an mNeonGreen-ParB focus) also migrated to the opposite cell pole, reaching its destination at 18 ± 2 and 17 ± 2 min, respectively, after *oriC* duplication (Videos S4 to S6 at the URL mentioned above; Table 2). Meanwhile, the DnaN-mCherry focus started to move from the invasive pole and transiently followed the ParB focus, indicating the progression of mother chromosome replication (Fig. 5D and Fig. 6D; see also Fig. S3D; see Videos S4 to S6 at the URL mentioned above). The next steps in chromosome multiplication were related to the number of released daughter cells. When three progeny cells were produced during predation on S. enterica, DNA replication was reinitiated from the chromosome located at the invasive pole. The newly assembled replisomes (visible as a large DnaN-mCherry focus) (see Fig. 5E; see also Video S4 at the URL mentioned above) moved away from the invasive pole (Fig. 5F and G; see also Video S4 at the URL mentioned above), and meanwhile, the first round of DNA replication was completed (indicated by disappearance of the first DnaN-mCherry signal) (Fig. 5G; see also Video S4 at the URL mentioned above). It was challenging to analyze the later stages of chromosome replication because in most cases it was unclear whether the observed foci were from transiently splitting sister replication forks and/or from temporally overlapping replication rounds. When four daughter cells were formed in an S. enterica bdelloplast, we observed two asynchronous reinitiation events; in both cases, new replisomes were assembled at the invasive pole (see Fig. S3H and I; see also Video S5 at the URL mentioned above). Notably, the first reinitiation of DNA replication started (Fig. S3H) after the disappearance of the first DnaN-mCherry focus, which was located in the middle of the filament and originated from the first initiation.

10.1128/mbio.00772-23.6 FIG S3 Dynamics of chromosome replication and oriC segregation in a B. bacteriovorus cell during proliferation in S. enterica—formation of four daughter cells. (A) Attachment of B. bacteriovorus to a S. enterica cell. (B) Bdelloplast formation; time = 0 min. (C) Appearance of the first DnaN-mCherry signal at the invasive pole—initiation of chromosome replication. (D to G) Replisome movement behind the ParB focus, indicating the progress in DNA multiplication. The appearance of second DnaN-mCherry foci, which presumably reflect splitting two replication forks and next merge in the middle of filament. (H and I) Two reinitiations of DNA replication from the mother chromosome located at the invasive pole and migration of both replisomes toward the midcell position. At the same time appearance of the third signal of ParB, which was located in the middle of the filament. (J) The disappearance of one replisome located nearest the old flagellar pole—termination of one chromosome replication and appearance of fourth mNeonGreen-ParB foci, which was located between signals at the invasive pole and in the middle of predator cell, respectively. (K) Termination of chromosomes replication and formation of four daughter cells. Photos represent merged bright-field and fluorescence (red and green) images. The B. bacteriovorus cell and the bdelloplast are marked by yellow and blue dotted lines, respectively. Scale bar = 1 μm. The full time-lapse is shown in Video S5 at https://figshare.com/authors/Jolanta_Zakrzewska-Czerwinska/14854612. Download FIG S3, TIF file, 7.4 MB. Copyright © 2023 Pląskowska et al. 2023 Pląskowska et al. https://creativecommons.org/licenses/by/4.0/ This content is distributed under the terms of the Creative Commons Attribution 4.0 International license.

In S. flexneri bdelloplasts, we observed up to five DnaN-mCherry foci, indicating that three (or more) pairs of replication forks were simultaneously active in a single filament. Unexpectedly, in an S. flexneri bdelloplast producing five (or more) progeny cells, at later stages of chromosome multiplication (185 min after bdelloplast formation) (Fig. 6F; see also Video S6 at https://figshare.com/authors/Jolanta_Zakrzewska-Czerwinska/14854612), we noticed the appearance of a DnaN-mCherry focus at the former flagellar pole, suggesting that replication may also initiate at the opposite pole.

In all bacteria, replication initiation is synchronized with the segregation of newly synthesized *oriC* region(s) (24). In all of the preys analyzed in our study, shortly after initiation of the first replication round, one of the newly synthesized *oriC* regions visible as a ParB focus (2nd ParB focus) moved toward the former flagellar pole (Fig. 3, 5, and 6 and Table 2; see also Fig. S3; see Videos S2 and S4 to S6 at https://figshare.com/authors/Jolanta_Zakrzewska-Czerwinska/14854612). In S. enterica or S. flexneri, similar to E. coli (3), the number of ParB foci gradually increased as new copies of the *oriC* region were synthesized (Table 2). The second round of chromosome segregation started at 74 ± 5 min after the first round in S. enterica and at 69 ± 5 min in S. flexneri (*n* = 100, data not shown). The newly assembled segrosome (3rd ParB focus) also migrated toward the opposite pole but did not reach the end of the filament, which was already occupied by the 2nd ParB complex; the 3rd ParB focus remained at the midcell (Fig. 5 and 6; see also Fig. S3; see Videos S4 to S6 at the URL mentioned above). After initiation of the third round of replication, the newly formed ParB focus (4th ParB focus) moved away from the invasive pole to its final location between the 1st and 3rd ParB foci (Fig. 6F and Table 2; see also Fig. S3J). Careful examination confirmed that the number of ParB foci reflected the number of released daughter cells (*n* = 300).

In conclusion, the choreography of B. bacteriovorus chromosome replication occurring in S. flexneri differs from that occurring in P. mirabilis and S. enterica; in S. flexneri, DNA replication may be reinitiated from the chromosome located at the former flagellar pole.

### Septation.

In all living cells, chromosome replication must be synchronized with cell division. Since chromosome replication is not followed immediately by cell division in B. bacteriovorus except in cases of binary fission (see below), we decided to elucidate how chromosome replication is synchronized with filament septation during the growth of B. bacteriovorus in preys that produce different numbers of daughter cells. For this purpose, we used a B. bacteriovorus strain producing DnaN-mCherry and FtsZ-mNeonGreen fusion proteins (Table 1). FtsZ filaments assemble into a ring (called the Z-ring) at the future site of the septum, and the appearance of an FtsZ focus is commonly regarded as an early marker of bacterial cell division (25). FtsZ from B. bacteriovorus is highly similar to other FtsZ homologs (53% identity with E. coli FtsZ) within the N-terminal domain, which contains conserved residues responsible for GTP binding, but possesses a unique C-terminal addition of ~160 amino acids (see Fig. S4 in the supplemental material).

10.1128/mbio.00772-23.7 FIG S4 Sequence alignment of selected FtsZ proteins. Protein sequences of 10 strains were aligned using the T-Coffee algorithm (Clustal W version 1.83) with default parameters and visualized using MView (version 1.63). FtsZ protein sequences were taken from the UniProt database (date of access, 9 November 2022). Download FIG S4, TIF file, 1.7 MB. Copyright © 2023 Pląskowska et al. 2023 Pląskowska et al. https://creativecommons.org/licenses/by/4.0/ This content is distributed under the terms of the Creative Commons Attribution 4.0 International license.

**FIG 7:**
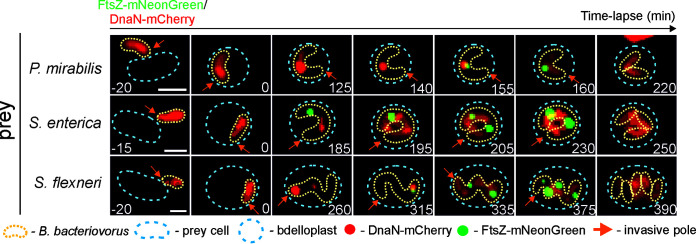
Replisomes dynamics in relation to cell division in a B. bacteriovorus cell growing inside different preys. Localization of replisome(s) (red) and divisiome(s) (i.e., FtsZ) (green) in a B. bacteriovorus cell growing inside P. mirabilis, S. enterica, and S. flexneri. Time = 0 min; bdelloplast formation. Photos represent merged bright-field and fluorescence (red and green) images. Scale bar = 1 μm. The full time-lapse is shown in Video S7 to S9 at https://figshare.com/authors/Jolanta_Zakrzewska-Czerwinska/14854612.

As expected, during predation in P. mirabilis, only one FtsZ signal appeared before the B. bacteriovorus filament divided into two daughter cells. Recruitment of FtsZ-mNeonGreen to the division site started 165 ± 4 min (*n* = 100) after bdelloplast formation (Table 3 and Fig. 7; see also Fig. S5A.G in the supplemental material). The FtsZ focus was localized in the middle of the mother cell (Fig. 7; Fig. S5A and B; see also Video S7 at https://figshare.com/authors/Jolanta_Zakrzewska-Czerwinska/14854612), and was visible for 49 ± 3 min (*n* = 100) (see Table 3). After FtsZ disassembly, the mother cells divided into two daughter cells. The duration of the B. bacteriovorus life cycle (from bdelloplast formation to progeny cell release) in P. mirabilis lasted approximately 4 h (Table 2).

**TABLE 3:** Comparison of the average times at which FtsZ foci appeared in B. bacteriovorus cells during the septation process in different preys

Prey	Avg time^*a*^ (min)				
	Appearance of FtsZ foci			Duration of FtsZ foci	Duration of life cycle
	1st^*b*^	2nd	3rd		
P. mirabilis	165 ± 4 (12 ± 2)			49 ± 3	252 ± 7
S. enterica	217 ± 5 (16 ± 2)	225 ± 6	244 ± 6	52 ± 6	288 ± 7
S. flexneri ^*c*^	310 ± 9 (19 ± 2)	ND	ND	ND	379 ± 11

a Average times were obtained after statistical analysis performed on 100 predator cells for each prey. For all measured values 95%, confidence interval was calculated, assuming a normal distribution of results. The appearance of fluorescent focus was measured from the bdelloplast formation (time zero). ND, not determined.

b In the parentheses, appearance time of the first FtsZ focus before the end of DNA replication.

c In the B. bacteriovorus cell growing inside S. flexneri, 4 to 7 of FtsZ-mNeonGreen foci were observed, but due to the formation of long, twisted filaments in this prey, the microscopic observation of FtsZ foci distribution was impossible.

10.1128/mbio.00772-23.8 FIG S5 Spatiotemporal analysis of chromosome replication and filament septation in a B. bacteriovorus cell growing in P. mirabilis. (A) The localization of replisomes (red) and divisome (green) in a predatory cell B. bacteriovorus growing inside the P. mirabilis bdelloplast. (A) Attachment of B. bacteriovorus to P. mirabilis cell. (B) Bdelloplast formation; time = 0 min. (C) Assembly of the first replisome at the invasive pole of B. bacteriovorus cell—the start of chromosome replication. (D to F) Further steps of chromosome replication; splitting of replication forks (the appearance of the second DnaN-mCherry focus at the “flagellated” pole) and merging of replication forks at the midcell. (G) The appearance of FtsZ-mNeonGreen focus, which colocalized with the merged replisomes. (H and I) Termination of predatory chromosome replication (disassembly of replisomes) and filament division into two progeny cells. (B) Localization of divisome in relation to filament length in B. bacteriovorus. Subcellular localization of FtsZ-mNeonGreen foci was analyzed in cells (*n* = 100) when the strongest fluorescent signal was observed. Photos represent merged bright-field and fluorescence (red and green) images. The B. bacteriovorus cell and the bdelloplast are marked by yellow and blue dotted lines, respectively. Scale bar = 1 μm. The full time-lapses are shown in Video S7 at https://figshare.com/authors/Jolanta_Zakrzewska-Czerwinska/14854612. Download FIG S5, TIF file, 0.5 MB. Copyright © 2023 Pląskowska et al. 2023 Pląskowska et al. https://creativecommons.org/licenses/by/4.0/ This content is distributed under the terms of the Creative Commons Attribution 4.0 International license.

Investigation of the septation process during predation on S. enterica revealed that two or three FtsZ signals were usually visible inside a single filament (Fig. 7; see also Fig. S6 in the supplemental material; see Video S8 at https://figshare.com/authors/Jolanta_Zakrzewska-Czerwinska/14854612), and, after their disappearance, the filament septated into three (see Fig. 7; Fig. S6) or four daughter cells, respectively. Surprisingly, in filaments growing in a prey producing three or more sibling cells, the FtsZ foci appeared asynchronously (for details, see Fig. 7 and Table 3; Fig. S6 and S7 in the supplemental material). For example, in S. enterica producing four daughter cells, the first FtsZ focus was visible 217 ± 5 min after bdelloplast formation while the last one (i.e., the third one) appeared approximately half an hour later (244 ± 6 min after bdelloplast formation) (Table 3). The FtsZ foci remained noticeable for 52 ± 6 min and then underwent disassembly, and the filament then divided into four daughter cells. Interestingly, the FtsZ foci are disassembled almost synchronously; in most analyzed filaments (>90%) dividing into 3 or 4 daughter cells, the FtsZ foci (i.e., 2 or 3, respectively) disappeared within 5 min (see Table S3 at the URL mentioned above).

10.1128/mbio.00772-23.9 FIG S6 Replisome dynamics in relation to divisomes position during the cell cycle in B. bacteriovorus cell growing inside S. enterica and creating three progeny cells. Time-lapse analysis of DnaN-mCherry and FtsZ-mNeonGreen signals localization during the cell cycle in B. bacteriovorus undergoes in S. enterica. (A) Attachment of B. bacteriovorus to a S. enterica cell. (B) Bdelloplast formation; time = 0 min. (C) Appearance of the first DnaN-mCherry focus at the invasive pole of B. bacteriovorus cell—initiation of chromosome replication. (D) Migration of DnaN-mCherry signal towards the pilus pole. (E and F) Reinitiation of DNA replication from the mother chromosome located at the invasive pole and migration of both replisomes toward the opposite pole. (G) The disappearance of one replisome located near “flagellated” pole—termination of multiplication of one chromosome. At the same time and localization appearance of the first FtsZ-mNeonGreen foci. (H to J) Termination of replication process (disassembly of replisomes) and appearance of the second divisome. (K) Termination of septation process with formed three daughter cells. Photos represent merged bright-field and fluorescence (red and green) images. The B. bacteriovorus cell and the bdelloplast are marked by yellow and blue dotted lines, respectively. Scale bar = 1 μm. The full time-lapse is shown in Video S8 at https://figshare.com/authors/Jolanta_Zakrzewska-Czerwinska/14854612. Download FIG S6, TIF file, 7.6 MB. Copyright © 2023 Pląskowska et al. 2023 Pląskowska et al. https://creativecommons.org/licenses/by/4.0/ This content is distributed under the terms of the Creative Commons Attribution 4.0 International license.

10.1128/mbio.00772-23.10 FIG S7 Spatiotemporal analysis of representative B. bacteriovorus cell showing the localization of replisomes (red) and divisomes (green) in a predatory cell growing inside the S. flexneri bdelloplast. (A) Attachment of B. bacteriovorus to S. flexneri cell. (B) Bdelloplast formation; time = 0 min. (C) Assembly of the first replisome at the invasive pole—the beginning of multiplication DNA. (D to F) Migration of the first replisome toward the opposite pole and appearance of another DnaN-mCherry signals from the invasive (reinitiation of DNA replication). (G) Reinitiation of DNA replication from the chromosome located at the “flagellated” pole and appearance of the first FtsZ-mNeonGreen foci localized at the same position as the last replisome. (H and I) Termination of DNA replication (disassembly of replisome) and appearance of another divisomes. (J) Termination of septation process and division into five progeny cells. Photos represent merged bright-field and fluorescence (red and green) images. The B. bacteriovorus cell and the bdelloplast are marked by yellow and blue dotted lines, respectively. Scale bar = 1 μm. The full time-lapse is shown in Video S9 at https://figshare.com/authors/Jolanta_Zakrzewska-Czerwinska/14854612. Download FIG S7, TIF file, 7.2 MB. Copyright © 2023 Pląskowska et al. 2023 Pląskowska et al. https://creativecommons.org/licenses/by/4.0/ This content is distributed under the terms of the Creative Commons Attribution 4.0 International license.

In the case of B. bacteriovorus growing in S. flexneri, it was difficult to precisely analyze the septation process, as the long, twisted filaments formed in this prey prevented in depth microscopic analysis. At the beginning of the septation process, we were able to observe the sequential appearance of multiple FtsZ signals (up to 7) and, as in other preys, the FtsZ rings never formed simultaneously (see, for example, Fig. S7; see also Video S9 at https://figshare.com/authors/Jolanta_Zakrzewska-Czerwinska/14854612).

Moreover, the TLFM analysis revealed that the recruitment of FtsZ to the Z ring started before the end of DNA synthesis. In all preys, the first FtsZ-mNeonGreen focus was visible approximately 10 to 20 min before the disassembly of the last replisome (Table 3 and Fig. 7; see also Videos S7 to S9 at https://figshare.com/authors/Jolanta_Zakrzewska-Czerwinska/14854612), namely, at 12 ± 2 min in P. mirabilis (85%, *n* = 100), 16 ± 2 min in S. enterica (88%, *n* = 100), and 19 ± 2 min in S. flexneri (90%, *n* = 100).

At the end of DNA synthesis, in binary dividing cells, the FtsZ-mNeonGreen focus consistently colocalized with DnaN-mCherry at the midcell position (*n* = 100). This is simultaneously the junction point of the two replication forks (see the overlapped FtsZ-mNeonGreen and DnaN-mCherry signals in Fig. 7, Fig. S5 in the supplemental material, and Video S7 at https://figshare.com/authors/Jolanta_Zakrzewska-Czerwinska/14854612). Interestingly, when three daughter cells were produced in an S. enterica bdelloplast, the first FtsZ focus was visible near the former flagellar pole (Fig. 7), where the first round of replication and segregation had already been completed (see Fig. 7 and Table 3; see also Fig. S6). Meanwhile, at the end of the second round of replication, the DnaN focus was located asymmetrically with respect to the filament length. It was closer to the invasive pole (65%, *n* = 100).

In S. flexneri, we were unable to precisely monitor the position of FtsZ rings in relation to the last visible replisome, especially in long filaments. However, we noticed that in a filament that would divide into five progeny cells, the first FtsZ-mNeonGreen focus frequently colocalized with the last visible DnaN-mCherry focus (78%, *n* = 100) (Fig. 7; see also Fig. S7G), which was positioned close to the former flagellar pole (consistent with observations in S. enterica). We also observed that in S. flexneri producing five (or more) progeny cells, the initiation of replication may also take place at the former flagellar pole (at later stages of chromosome multiplication) (see Fig. 6F, 185 min after bdelloplast formation; see also Video S6 at https://figshare.com/authors/Jolanta_Zakrzewska-Czerwinska/14854612).

Since we found that the formation of FtsZ rings in the predatory filament is an asynchronous process and the number of released B. bacteriovorus cells depends on the prey/cell size, we decided to analyze the size of the progeny cells released from the analyzed pathogens in comparison to the model prey, E. coli. Length measurement of mature B. bacteriovorus cells after bdelloplast lysis showed that there were statistically relevant differences (see Fig. S8 at https://figshare.com/authors/Jolanta_Zakrzewska-Czerwinska/14854612). The average lengths of the generated B. bacteriovorus cells were 1.25 ± 0.03 μm for P. mirabilis, 1.16 ± 0.03 μm for S. enterica, 1.35 ± 0.03 μm for S. flexneri, and 1.11 ± 0.04 μm for E. coli. On average, the longest progeny cells were released from S. flexneri (usually 5 or 6 progeny cells), which had the largest cell size of the analyzed preys. The daughter cells born from P. mirabilis (2 progeny cells) were longer than those released from E. coli (usually 3 or 4 progeny cells) and S. enterica (usually 3 or 4 progeny cells). There is, therefore, no direct correlation between the number of progeny cells and the size of the progeny cells.

Understanding the proliferation of B. bacteriovorus in different pathogens may also be helpful for its use to efficiently eliminate a given pathogen, i.e., by calculating the optimal ratio between the predator and the prey. We developed a new application to simulate these infection dynamics using the number of predatory and prey cells, the average number of progeny cells released from a single prey cell (Fig. 1), and the duration of the reproductive phase (Table 2). For the purposes of our simulation, we assume that all released progeny cells immediately find new preys and that prey cells do not replicate (similar to the killing curves where prey cells were resuspended in Ca-HEPES buffer). The surviving number of prey cells is calculated as n_prey_i+1_ = n_prey_i_ − n_bdellovibrio_i_ and the new number of *Bdellovibrio* cells is calculated as n_bdellovibrio_i+1_ = n_bdellovibrio_i_ × n_progeny. This simulation, called BdelloSim, allowed us to calculate the time necessary to eliminate the entire prey population given the same starting population sizes of prey and B. bacteriovorus cells as those used for the killing curves (see Fig. S1 in the supplemental material; see also Fig. S9 at https://figshare.com/authors/Jolanta_Zakrzewska-Czerwinska/14854612; and Materials and Methods). The time required for the complete lysis of prey (see Fig. S9 at the URL mentioned above) obtained by BdelloSim are consistent with the killing curves, for P. mirabilis, 2,500 min; for S. enterica, 1,740 min; and for S. flexneri, 1,520 min.

Taken together, the results of our TLFM analyses revealed the cell cycle parameters of B. bacteriovorus filaments growing in three different pathogens. Our work demonstrates that the mode of the B. bacteriovorus cell cycle depends on the prey cell size, with filaments proliferating in small cells, such as P. mirabilis, dividing by binary fission, while those growing in larger prey cells undergo nonbinary fission.

## DISCUSSION

**FIG 8:**
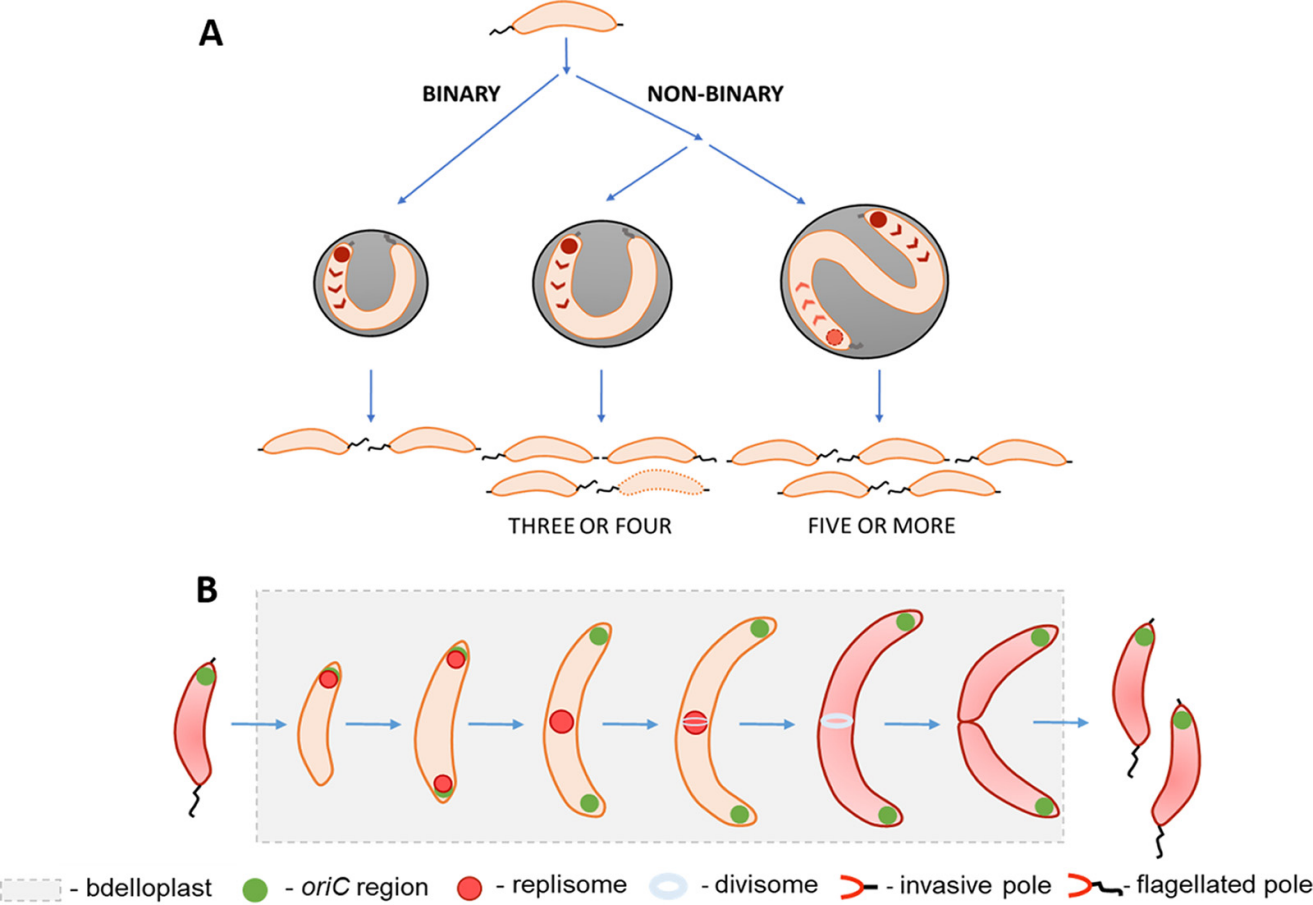
Binary and nonbinary proliferation of Bdellovibrio bacteriovorus in preys differing in size. (A) Choreography of DNA replication initiation and modes of cell division. B. bacteriovorus depending on a prey size divides either by binary (in small prey cells) or by nonbinary fission (in larger prey cells) producing two or more daughter cells, respectively. The chromosome replication, regardless of the mode of cell division, is always initiated at the invasive pole. In larger preys (when 5 or more progeny cells are formed), the last round(s) of chromosome replication may also be initiated at the cell pole, which is opposite to the invasive pole. (B) Cell cycle of B. bacteriovorus dividing by binary fission, highlighting the dynamics of chromosome replication and cell division. The chromosome replication is initiated at the invasive pole and then one of the newly replicated *oriC* regions is segregated toward the flagellated pole. During the progress of DNA replication, the replication forks split (one replisome follows the segregated *oriC* region) and merge at the midcell and then disassembled. The FtsZ ring assembly is initiated at the midcell before the termination of DNA replication, and next the filament is divided into two progeny cells.

The use of predatory bacteria such as B. bacteriovorus is currently regarded as a promising strategy to kill antibiotic-resistant pathogens (9, 26, 27). Therefore, understanding the mode and dynamics of B. bacteriovorus proliferation in various dangerous pathogens may facilitate knowledge-based improvements in B. bacteriovorus strains for use as a “living antibiotic” in human and veterinary medicine, particularly to combat multidrug-resistant pathogens. Previously, B. bacteriovorus was believed to undergo a nonbinary life cycle. In this study, we demonstrate for the first time that this predatory bacterium proliferates by either a binary or a nonbinary mode of fission, depending on the prey and its size (Fig. 8A). We also show that the choreography of DNA replication in this system exhibits unique features and is related to the mode of fission giving rise to different number of progeny cells (Fig. 8A). Finally, we show that FtsZ rings are assembled asynchronously in nonbinary proliferating cells of B. bacteriovorus.

### B. bacteriovorus undergoes binary fission when growing in a small prey cell.

Our data reveal that, in small pathogenic bacteria such as P. mirabilis (cell length, <2 μm), only two predatory cells are formed (see Fig. 8A and B). In this prey, the FtsZ ring is assembled in the middle of the filament and then undergoes disassembly, whereupon the filament is divided into two daughter cells. Interestingly, the assembly of the FtsZ ring begins before the termination of DNA replication. Detailed microscopic analysis of P. mirabilis undergoing predation by B. bacteriovorus reveals that the old pole that had been flagellated becomes an invasive pole with a visible ParB complex. Simultaneous analysis of both replication and segregation processes confirmed this observation: during ongoing DNA replication, one of the newly replicated *oriC* regions (observed as a ParB focus) migrates toward the opposite pole (the one bearing a flagellum before the prey attack). Further microscopic observations demonstrated that, after B. bacteriovorus division, the released daughter cell with the invasive pole arising from the former flagellar pole of the mother cell attaches to the next cell prey using this cell pole and proliferates within this prey. Thus, after the division by binary fission, one daughter cell inherits the old invasive pole while the other inherits the former flagellar pole of the mother cell, which must be extensively rebuilt to become an invasive pole. Consequently, flagella must be formed at new poles emerging from the last division (Fig. 8).

Collectively, our results demonstrate for the first time that B. bacteriovorus may undergo binary fission and P. mirabilis provides a simple and ideal model prey for studying the life cycle of this predatory bacterium.

### Unique choreography of chromosome replication in binary-dividing B. bacteriovorus.

As in other preys, including E. coli (3, 19), chromosome replication in B. bacteriovorus undergoing predation on P. mirabilis starts at the invasive pole. Then, shortly after the initiation of DNA synthesis, the replisomes split; one migrates toward the former flagellar pole while the other remains at the invasive pole. Before the end of DNA synthesis, the two replisomes move toward each other to meet at the cell center, where assembly of the FtsZ ring has begun. Thus, at the beginning of chromosome replication, the choreography of this process in B. bacteriovorus resembles that found in other asymmetrically dividing bacteria, such as C. crescentus (28) and Vibrio cholerae (chromosome I) (29), where the replisomes are also assembled at a cell pole. This is contrary to chromosome replication in E. coli (24, 30) and B. subtilis (31), where replication starts at the midcell. However, the spatiotemporal choreography of further steps of B. bacteriovorus chromosome replication differs from those described in other asymmetrically dividing bacteria. For example, in C. crescentus, the replisomes migrate together from the cell pole to the midcell position, where replication is terminated (28, 32).

The assembly of replisomes at the invasive pole of B. bacteriovorus (3, 19) suggests that, as in other asymmetric bacterial cells, the *oriC* region must be anchored to the cell pole via a specific polar protein(s). To date, such polar proteins, also called “hub” proteins, have been characterized in C. crescentus (PopZ) (33), V. cholerae (HubB) (34), and *Streptomyces* (DivIVA) (35, 36). The B. bacteriovorus genome lacks homologs of PopZ or HubP but does encode a version of DivIVA (37). It was recently speculated that DivIVA together with a recently identified homolog of the filament-forming protein, bactofilin (37), may be involved in maintaining the polarity of this predatory bacterium. However, the precise role of these proteins in anchoring the B. bacteriovorus *oriC* region at the invasive pole remains to be elucidated. We cannot exclude the possibility that other proteins function to anchor the chromosome at the invasive pole.

In summary, we herein present, for the first time, the choreography of chromosome replication and cell division during the binary cell cycle of B. bacteriovorus (see Fig. 8B).

### FtsZ rings are asynchronously assembled across the elongated filament during nonbinary fission.

In larger preys (cell length, >2 μm), the filament of B. bacteriovorus undergoes nonbinary division and odd or even numbers of cells are produced. As expected, the number of released progeny cells strictly depends on the number of FtsZ rings that are formed before filament division (Fig. 7). Although the septation process in B. bacteriovorus was previously shown to be synchronous (4), our study revealed that FtsZ is recruited asynchronously to the sites of division, with the first FtsZ signal appearing before the end of DNA synthesis (Fig. 7). Further FtsZ signals appear sequentially. For example, in a filament that will divide into four nascent cells, the third FtsZ signal is assembled ~30 min after the appearance of the first FtsZ signal (Table 3). However, the disassembly of FtsZ rings was found to be a synchronous process (see Table S3 at https://figshare.com/authors/Jolanta_Zakrzewska-Czerwinska/14854612), which is consistent with previous studies showing a synchronous septation in B. bacteriovorus (4). In *Streptomyces*, which is a Gram-positive filamentous bacterium, the multigenomic filaments also undergo synchronous division to form chains of unigenomic exospores, but, in contrast to B. bacteriovorus, the FtsZ rings are synchronously assembled in this case (5, 38), and this occurs after the cessation of DNA replication (39). Thus, we speculate that in B. bacteriovorus, yet undiscovered replication checkpoints act to coordinate chromosome replication with cell cycle progression, such as by tuning FtsZ ring placement to prevent the formation of daughter cells with a missing/guillotined chromosome or an additional copy of the chromosome.

### In nonbinary dividing long filaments, the initiation of chromosome replication may also occur at the cell pole opposite the invasive pole.

Similar to the results obtained by Laloux’s group (3), we observed that DnaN foci usually appeared sequentially at the invasive pole, suggesting that there is asynchronous firing of replication initiation from the chromosome attached to this pole (the newly appeared fluorescently tagged DnaN foci colocalized with the ParB attached to the invasive pole) (Fig. 5 and 6; see also Fig. S3 in the supplemental material). However, it proved hard to follow the further progression of chromosome multiplication (especially in filaments that would divide into several or more nascent cells) because it was difficult to distinguish between transiently splitting sister replication forks and temporally overlapping replication rounds. In *Streptomyces* vegetative hyphae, DNA replication is also (re)initiated from the chromosome that is anchored at the filament tip, and one of the newly replicated *oriC* regions bound by ParB protein is unidirectionally segregated (6). Thus, in both B. bacteriovorus and *Streptomyces*, after *oriC* duplication, ParB complexes must be reestablished at both daughter *oriC*s at the cell pole; thereafter, one of them is captured by apically localized hub proteins and becomes abandoned by the segregation machinery.

Surprisingly, during the proliferation of B. bacteriovorus in S. flexneri, we noticed that the reinitiation of chromosome replication may also occur at the former flagellar pole (Fig. 6; see also Video S6 at https://figshare.com/authors/Jolanta_Zakrzewska-Czerwinska/14854612). This phenomenon was observed only in the very late stages of chromosome multiplication at ~160 min after the first initiation of chromosome replication. We speculate that in the longer filaments (those that will divide into five or more progeny cells), the former flagellar pole is converted to an invasive pole while chromosome replication is still in progress. Therefore, the last round(s) of DNA replication may also be initiated from the chromosome that is already attached to the newly formed invasive pole. However, further work is needed to elucidate the mechanism(s) underlying the molecular switch that activates replication initiation from the chromosome where it attaches to the old invasive pole and/or the pole that has been converted from former flagellar to invasive.

In summary, our findings demonstrate that in a nonbinary dividing filament of B. bacteriovorus, chromosome replication may be initiated not only from the invasive pole, but also from the opposite pole, which has presumably been converted to an invasive pole.

### Conclusions.

We herein set the scene for B. bacteriovorus to become a novel model that enables the study of both binary and nonbinary cell division. Our findings provide new insight into the life cycle of a predatory bacterium that exhibits bimodal fission and demonstrate that the mode of division depends on the size of the prey bacterium inside which B. bacteriovorus grows. Further studies on the B. bacteriovorus life cycle should aim to shed light on the regulatory mechanisms responsible for switching between the binary and nonbinary modes of the cell cycle. Moreover, understanding the proliferation of B. bacteriovorus in different pathogens may facilitate its use to efficiently eliminate a given pathogen (i.e., by calculating the optimal ratio between the predator and the prey).

## MATERIALS AND METHODS

### Bacterial strains and culture conditions.

The culture conditions and construction of bacterial strains were as described in Makowski et al. (19). Briefly, the plasmids used to construct B. bacteriovorus HD100 strains were propagated in E. coli DH5α grown in LB broth or on LB agar plates (supplemented with 50 μg/mL kanamycin) and then transformed into E. coli S17-1. The E. coli strains used as prey for B. bacteriovorus were liquid cultured in YT medium (0.8% Bacto tryptone, 0.5% yeast extract, 0.5% sodium chloride, pH 7.5) with (S17-1 pZMR100) or without (S17-1, ML35) kanamycin (50 μg/mL) at 37°C with shaking (180 rpm). B. bacteriovorus cells were grown by predation on E. coli (ML35, S17-1, or S17-1 pZMR100) or pathogenic preys (P. mirabilis, S. enterica, or S. flexneri) in Ca-HEPES buffer (25 mM HEPES, 2 mM calcium chloride, pH 7.6). All pathogenic bacteria were grown in LB broth at 37°C with shaking (180 rpm) or on LB agar plates at 37°C. Details regarding the strains, plasmids and oligonucleotides used in this study are presented in Table S1 in the supplemental material.

### Predatory killing curves.

B. bacteriovorus strain HD100 (wild type) was propagated in P. mirabilis, S. enterica, or S. flexneri. An overnight culture of prey cells was spun down at 5,000 rpm for 10 min at 20°C, and the cells were washed with Ca-HEPES buffer and back diluted to an optical density at 600 nm (OD_600_) of 1.0 with Ca-HEPES buffer. The cultures of B. bacteriovorus cells were filtered through 0.45-μm filters, and 20 μL of filtered predator cells (2.1 × 10^7^ cells) was mixed with 280 μL of prey suspension (19). Lysis curves (40) were analyzed using Bioscreen C (Automated Growth Curves Analysis system; Growth Curves, USA). The decrease of optical density (OD_600_) at 30°C was measured at 20-min intervals for 42 h. Experiments were done in three independent biological replicates. The Weibull four-parameters model was fitted to the data using the drc R package to calculate Effective kill time 50% of the host’s cells (EKT_50_) values and their standard errors (41).

### DNA manipulation and construction of B. bacteriovorus HD100 strains.

DNA manipulations in E. coli were carried out using standard molecular methods (42). Oligonucleotides were synthesized by Sigma-Aldrich. Reagents and enzymes were supplied by Thermo Scientific, Sigma-Aldrich, and NEB. The following B. bacteriovorus HD100 strains were constructed: DnaN-mNeonGreen, DnaN-mCherry, mNeonGreen-ParB/DnaN-mCherry, and FtsZ-mNeonGreen/DnaN-mCherry. In them, the replisomes were labeled green or red by DnaN fusion or the segrosomes and divisomes were labeled green by ParB and FtsZ fusions, respectively (Table S1). In all constructed strains, the gene of interest (*dnaN*, *parB*, or *ftsZ*) was exchanged for a fusion gene that was located in the native chromosomal locus and expressed under its own native promoter(s) (see Table S1).

The coding sequences of the relevant B. bacteriovorus genes (*dnaN*, *parB*, or *ftsZ*) or reporter genes (*mNeonGreen* or *mCherry*) were amplified (all primers are listed in Table S1) using chromosomal DNA isolated from B. bacteriovorus HD100 or plasmids (pAKF220-mNeonGreen or p2Nil-lsr2-mCherry), respectively, as the templates. Gibson assembly was used to clone the PCR products into the pK18mobsacB plasmid. The obtained constructs (pK18dnaN-mNeonGreen, pK18dnaN-mCherry, pK18mNeonGreen-parB, and pK18ftsZ-mNeonGreen) were transformed into E. coli S17-1 and conjugated to B. bacteriovorus strain HD100 as described previously (19). Scarless allelic replacements of the wild-type chromosomal *dnaN*, *parB*, and *ftsZ* genes by the gene fusions were performed using two-step homologous recombination with suicide vector pK18mobsacB as described by Steyert and Pineiro (43). Double-crossover (DCO) events were performed on single-crossover (SCO) strains of B. bacteriovorus. SCO strains were grown for 2 days without antibiotics in Ca-HEPES and then for an additional 2 days in Ca-HEPES with 5% sucrose. The obtained mutants were diluted and grown on two-layer YPSC agar plates (1% Broadbeam peptone, 1% yeast extract, 0.5% sodium acetate, 0.25% magnesium sulfate [pH = 7.6]). The final concentrations of agarose in the YPSC plates were 1% in the bottom layer and 0.6% in the top layer. The plates were incubated at 30°C until plaques appeared. DCO strains were searched by plaque PCR screening. The obtained B. bacteriovorus strains harbored gene fusions under the control of the endogenous promoters. Proper construction of all fluorescent strains was verified by DNA sequencing, Western blotting (see Text S1), and fluorescence microscopic observations.

10.1128/mbio.00772-23.1 TEXT S1 Western blot analysis. Download Text S1, DOCX file, 0.01 MB. Copyright © 2023 Pląskowska et al. 2023 Pląskowska et al. https://creativecommons.org/licenses/by/4.0/ This content is distributed under the terms of the Creative Commons Attribution 4.0 International license.

### Microscopic analysis.

Agarose gel (1%) in Ca-HEPES buffer was poured onto a basic slide and allowed to solidify. Lysate (10 μL) of fresh prey (wild-type E. coli, P. mirabilis, S. enterica, or S. flexneri) was added in a layer of agarose gel, and the slide was sealed with a coverslip. Images were taken in phase-contrast mode using a Leica DM6 B microscope equipped with a 100×/1.32 OIL PH3 objective. Captured images were analyzed using the ImageJ Fiji suite (http://fiji.sc/Fiji).

### Time-lapse fluorescence microscopy.

Time-lapse fluorescence microscopy (TLFM) was performed on B04A plates with an ONIX flow control system (Merck-Millipore) (44) or on an agarose pad (45) with ibidi cell-imaging dishes (19). For both systems, an overnight stationary-phase culture of prey cells was diluted 25 times and loaded into the flow chamber. Next, B. bacteriovorus cells were injected to the flow chamber (pressure, 4 lb/in^2^) in Ca-HEPES buffer at 30°C for 2 min or until predatory cells were visible in the field of view. Agarose pads were prepared as described previously (17, 25) using P. mirabilis as a prey cell. In both methods, images were recorded every 5 min using a Delta Vision Elite inverted microscope equipped with an Olympus 100×/1.40 objective and a Cool SNAP HQ2-ICX285 camera. DnaN-mCherry was visualized by detection of mCherry (EX575/25; EM625/45) with an 80-ms exposure time and 50% intensity. Proteins fused with mNeonGreen were visualized by detection of green fluorescent protein (GFP) (EX475/28; EM525/48) with a 50-ms exposure time and 32% intensity. Bright-field images were taken with a 30-ms exposure time and 10% intensity. The captured images were analyzed using the ImageJ Fiji suite (http://fiji.sc/Fiji). TLFM experiments were done in three independent biological replicates.

### Statistical analysis and B. bacteriovorus growth simulation.

All obtained values and time points were calculated from at least one hundred B. bacteriovorus cells using three independent time-lapse microscopy experiments. We determined using CFU that an OD_600_ of 1.0 corresponds to 1.5 × 10^9^ for P. mirabilis, 9.5 × 10^8^ for S. enterica, and 3 × 10^8^ for S. flexneri. For all measured values, the 95% confidence interval (CI) was calculated, assuming a normal distribution of results. Simulations of B. bacteriovorus population growth were done using the R language based on the experimental data concerning the life cycle length and number of progeny cells. The code is available at https://github.com/astrzalka/BdelloSim, and the online version of the application, which allows for simultaneous analysis of three different preys, is available at http://microbesinwroclaw.biotech.uni.wroc.pl:3838/BdelloSim/.

TABLE S3, Fig. S8 and S9, and Videos S1 to S9 are available at https://figshare.com/authors/Jolanta_Zakrzewska-Czerwinska/14854612.
