# Supplementary material for: Binary or Nonbinary Fission? Reproductive Mode of a Predatory Bacterium Depends on Prey Size
Source: mBio. 2023 May 10;14(3):e00772-23. doi: 10.1128/mbio.00772-23 (PMC10294633; doi:10.1128/mbio.00772-23)
Supplement: TABLE S1 [file mbio.00772-23-s0002.docx]

**Table S1. Bacterial species, strains, plasmids, and primers used in this study.**

| **Oligonucleotides** | | |
| --- | --- | --- |
| ***B. bacteriovorus* strains** | **Name** | **Sequence 5’ to 3’** |
| HD100DnaN-mCherry | pK18_DnaN_fw | ACCTCCGGGTACCGAGCTCGATGAAATTAGAGATTGATAAGCGAGATCTGTTAAGT |
|  | linker_DnaN_rv | CCGCCGAGCCGATTCTCATTGGCATCACAACGCAG |
|  | linker+mCherry_fw | GGCTCGGCGGGCTCGGCGGCGGGCTCGGGCGAGTTCATGGTGAGCAAGGGCGAGG |
|  | recF-mCherry_rv | CGAAAATCATTACTTGTACAGCTCGTCCATGCC |
|  | dnaN_linker_fw | AATGAGAATCGGCTCGGCGGGCT |
|  | mCherry_RecF_fw | TGTACAAGTAATGATTTTCGAAAGACTGCGTCTGG |
|  | pK18_RecF_rv | CAGCTATGACCATGATTACGCTACTCAAGGATTTGGCCATCCTTGA |
|  | DnaN_upstream_fw | ATGGACTTAGGGGATTTGTTAGG |
|  | FP_sek_rv2 | TACCCTGTCCGTTCTCTCCG |
|  | DnaN_DCO_fw | ACTGTGGAAATAGCTGTGGAAGG |
|  | RecF_DCO_rv | TACGAGCAACTCATCCACCAGC |
| HD100DnaN-mNeon | pK18_DnaN_fw | ACCTCCGGGTACCGAGCTCGATGAAATTAGAGATTGATAAGCGAGATCTGTTAAGT |
|  | linker_DnaN_rv | CCGCCGAGCCGATTCTCATTGGCATCACAACGCAG |
|  | linker+mNeon_fw | GGCTCGGCGGGCTCGGCGGCGGGCTCGGGCGAGTTCATGGTTTCGAAAGGAGAGGAGG |
|  | RecF_mNeon_rv | CGAAAATCATTTACTTGTACAGCTCGTCCATGCC |
|  | dnaN_linker_fw | AATGAGAATCGGCTCGGCGGGCT |
|  | mNeon_RecF_fw | CTATAAGTGAATGATTTTCGAAAGACTGCGTCTGG |
|  | pK18_RecF_rv | CAGCTATGACCATGATTACGCTACTCAAGGATTTGGCCATCCTTGA |
|  | DnaN_upstream_fw | ATGGACTTAGGGGATTTGTTAGG |
|  | FP_sek_rv2 | TACCCTGTCCGTTCTCTCCG |
|  | DnaN_DCO_fw | ACTGTGGAAATAGCTGTGGAAGG |
|  | RecF_DCO_rv | TACGAGCAACTCATCCACCAGC |
| HD100mNeon-ParB | pK18_parB_fw | GTCGACTCTAGAGGATCCCCTTACTGCCATCCTTCTTTAAGCCTATCTACC |
|  | linker_parB_rv | GGGCGAGTTCATGTCTGATATTGCTGTAGAATCCTCAAACAAG |
|  | mNeon_linker_rv | GAACTCGCCCGAGCCCGCCGCCGAGCCCGCCGAGCCCTTATAGAGTTCATCCATACC |
|  | parA_mNeon_rv | GGGAATTTAAATGGTTTCGAAAGGAGAGGAGGATAATATG |
|  | parB_linker_fw | TATCAGACATGAACTCGCCCGAGCCC |
|  | mNeon_parA_fw | TCGAAACCATTTAAATTCCCCTTCTTATGCCATTTGTTCTG |
|  | pK18_parA_rv | CGAATTCGAGCTCGGTACCCTTGAATGTTCACGCAAAAAGGAGGACT |
|  | parB_zew_fw | TCTGAACCGTCCCCTCGAAGC |
|  | parA_wew_SCO_rv | AATCCATCTTCGAGTACGACAGC |
|  | parB_wew_fw | CGTACGACGAGCCACGATGG |
|  | parA_wew_rv | ACCAGTATGACTTCGTGATCATCG |
| HD100FtsZ-mNeon | linker_FtsZ_fw | GCCCGCCGAGCCTTCTTTATTCAGATCGAATCCTTGTTTCTTGCG |
|  | pK18_FtsZ-rv | TACGAATTCGAGCTCGGTACCCGGGATGTTTGAGTTGGAAGAAAATATCAATATCGGTG |
|  | IpxH_mNeon_fw | GGCTCGGCGGGCTCGGCGGCGGGCTCGGGCGAGTTCATGGTTTCGAAAGGAGAGGAGG |
|  | linker_mNeon_fw | GGCTCGGCGGGCTCGGCGGCGGGCTCGGGCGAGTTCATGGTTTCGAAAGGAGAGGAGG |
|  | IpxH_mNeon_fw | ACCAGGCTTCCACTACTTATAGAGTTCATCCATACCCATCACG |
|  | FtsZ_linker_rv | TCTGAATAAAGAAGGCTCGGCGGGCT |
|  | pK18_IpxH_fw | CCAGTGCCAAGCTTGCATGCCTGCATCATAAATCCTCCTCAGAAGGCAGA |
|  | mNeon_IpxH_rv | ACTCTATAAGTAGTGGAAGCCTGGTTCATATCCGACAT |
|  | FtsZ_downstream_SCO_fw | ATAGGTGCAGGTGTAAGAACGC |
|  | FP_sek_rv2 | ATGGCTCGTGAAGTCCTGAGC |
|  | FtsZ_downstream_fw | TTCCCATTGCGCTCTTCAGCG |
|  | FtsZ_DCO_upstream_rv | ATCGCTGGACGGTATCACC |
| **Plasmids** | | |
| **Name** | **Plasmid features** | **Reference** |
| p2Nil-*lsr2-mCherry* | Plasmid carrying mCherry coding sequence; Kan^R^ | Marta Kołodziej |
| pAKF220 | Plasmid carrying mNeonGreen coding sequence; Amp^R^ | (1) |
| pK18*mobsacB* | Suicide vector used for conjugation and recombination into *Bdellovibrio* genome; Kan^R^ | (2) |
| pK18_dnaN_mCherry | Derivative of pK18mobsacB containing fusion gene *dnaN-mCherry*; Kan^R^ | This study |
| pK18_dnaN_mNeon | Derivative of pK18mobsacB containing fusion gene *dnaN-mNeonGreen*; Kan^R^ | This study |
| pK18_mNeon_ParB | Derivative of pK18mobsacB containing fusion gene *mNeonGreen-parB*; Kan^R^ | This study |
| pK18_ftsZ_mNeon | Derivative of pK18mobsacB containing fusion gene *ftsZ-mNeonGreen*; Kan^R^ | This study |
| ***B. bacteriovorus* strains** | | |
| **Name** | **Description/sequence** | **Source** |
| HD100 | Wild-type strain | DSMZ50701 |
| HD100DnaN-mCherry | HD100 *dnaN::dnaN-mCherry* | This study |
| HD100DnaN-mNeon | HD100 *dnaN::dnaN-mNeonGreen* | This study |
| HD100mNeon-ParB/DnaN-mCherry | HD100 *parB::mNeonGreen-parB dnaN::dnaN-mCherry* | This study |
| HD100FtsZ-mNeon/DnaN-mCherry | HD100 *ftsZ::ftsZ-mNeonGreen-ftsZ dnaN::dnaN-mCherry* | This study |
| **Bacterial species** | | |
| **Name** | **Description** | **Source** |
| *E. coli* S17-1 | thi pro hsdR^-^ hsdM^+^ recA; harboring plasmid RP4-Tc::Mu-Kn::Tn7, used as donor for conjugation of plasmids into *Bdellovibrio* | (3) |
| *E. coli* S17-1 pZMR100 | S17-1 strain containing pZMR100 plasmid to confer Kan^r^ ; used as Kan^r^ prey for *Bdellovibrio* | (4) |
| *E. coli* ML35 | Strain routinely used as prey for *B. bacteriovorus* | ATCC43827 |
| *Proteus mirabilis* | Non-pathogenic strain used as prey for *Bdellovibrio* | PCM1098 |
| *Salmonella enterica* | Non-pathogenic strain used as prey for *Bdellovibrio* | PCM2550 |
| *Shigella flexneri* | Non-pathogenic strain used as prey for *Bdellovibrio* | PCM1936 |

1. Rendulic S, Jagtap P, Rosinus A, Eppinger M, Baar C, Lanz C, Keller H, Lambert C, Evans KJ, Goesmann A, Meyer F, Sockett RE, Schuster SC. 2004. A predator unmasked: life cycle of Bdellovibrio bacteriovorus from a genomic perspective. Science 303:689–692.
2. Schäfer A, Tauch A, Jäger W, Kalinowski J, Thierbach G, Pühler A. 1994. Small mobilizable multi-purpose cloning vectors derived from the Escherichia coli plasmids pK18 and pK19: selection of defined deletions in the chromosome of Corynebacterium glutamicum. Gene 145:69–73.
3. Simon R, Priefer U, Pühler A. 1983. A Broad Host Range Mobilization System for In Vivo Genetic Engineering: Transposon Mutagenesis in Gram Negative Bacteria. 9. Nat Biotechnol 1:784–791.
4. Rogers M, Ekaterinaki N, Nimmo E, Sherratt D. 1986. Analysis of Tn7 transposition. Mol Gen Genet 205:550–556.
