# Supplementary figures and images for: Binary or Nonbinary Fission? Reproductive Mode of a Predatory Bacterium Depends on Prey Size
Source: mBio. 2023 May 10;14(3):e00772-23. doi: 10.1128/mbio.00772-23 (PMC10294633; doi:10.1128/mbio.00772-23)

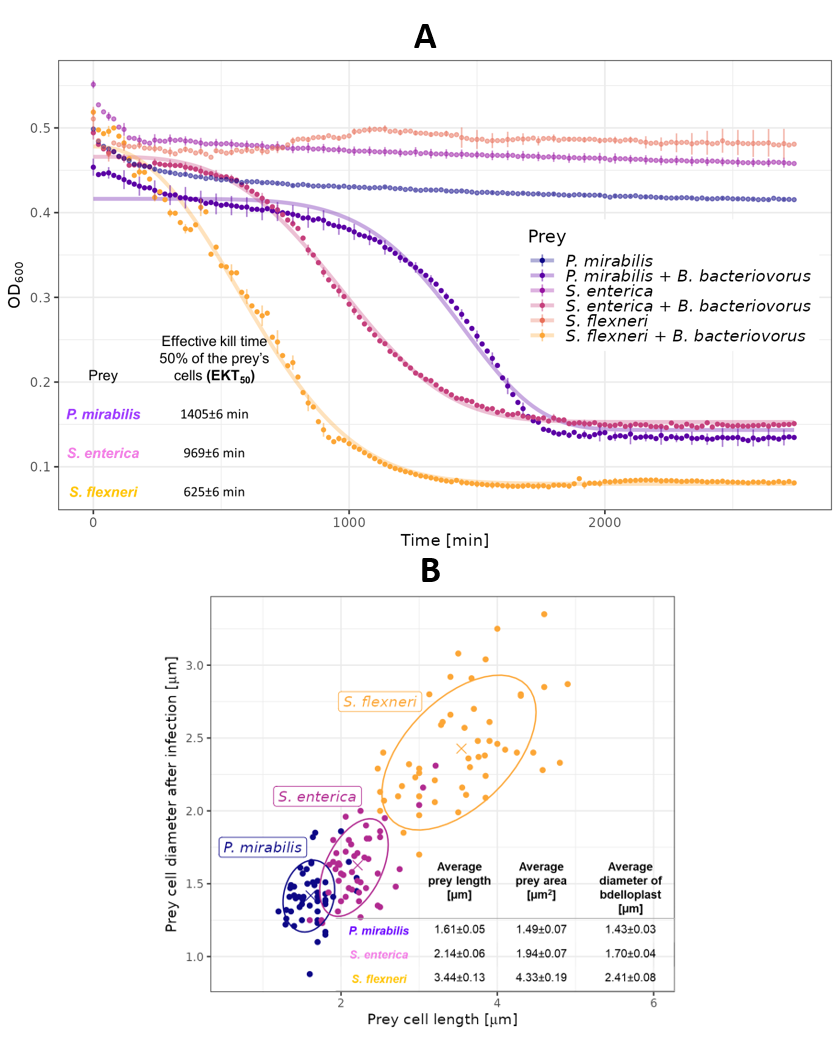

Supplement: FIG S1 [file mbio.00772-23-s0004.tif]

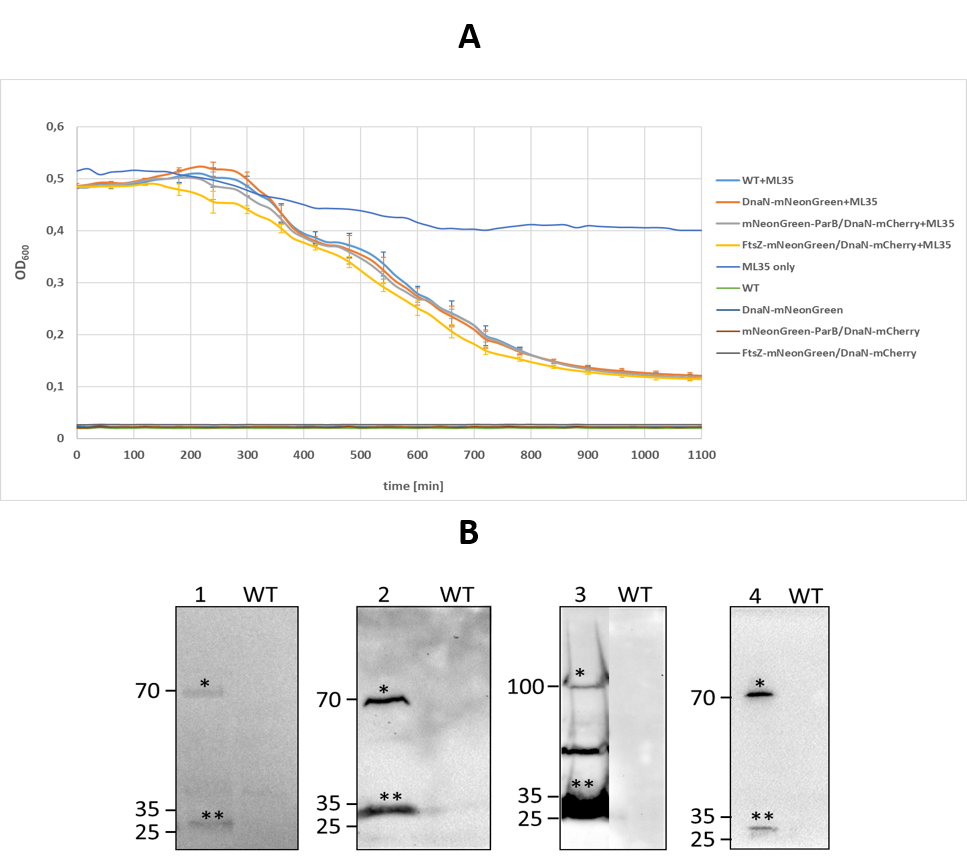

Supplement: FIG S2 [file mbio.00772-23-s0005.tif]

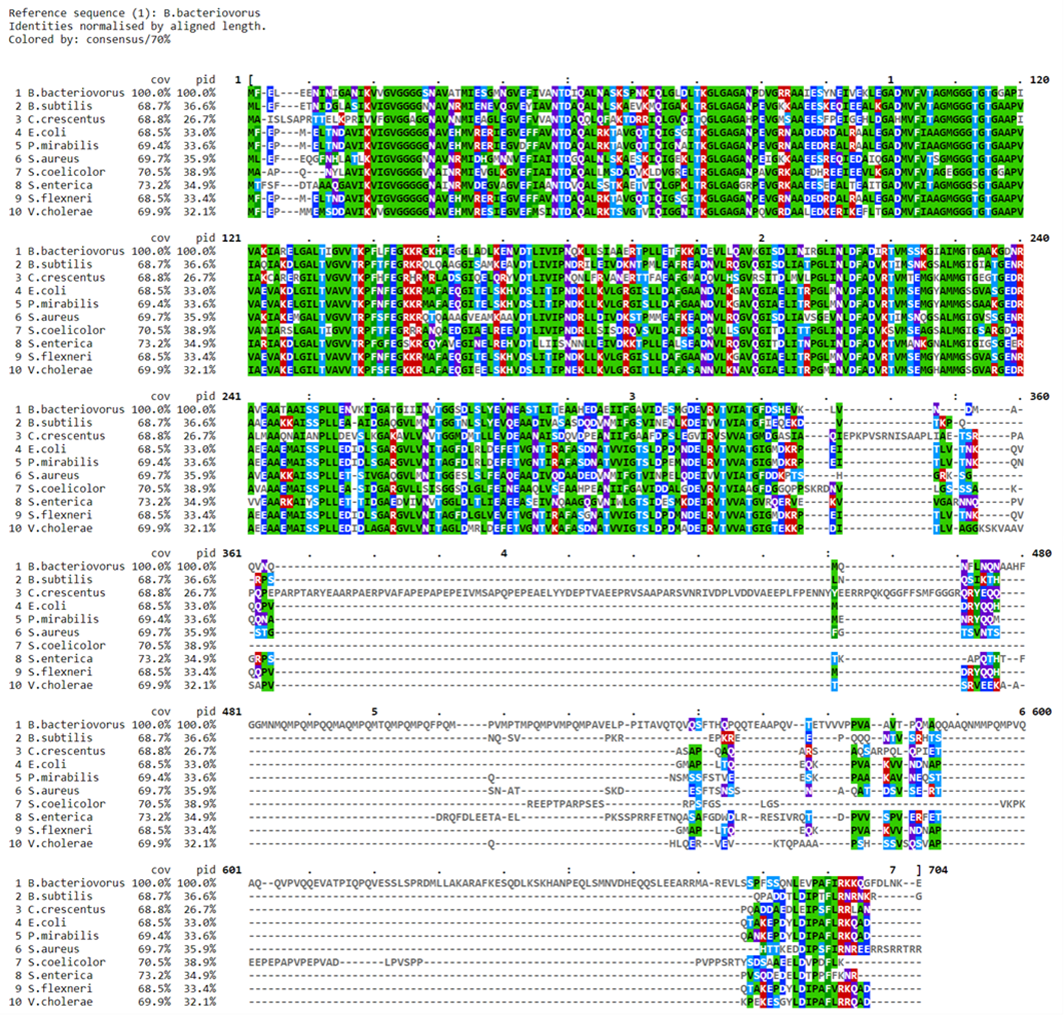

Supplement: FIG S4 [file mbio.00772-23-s0007.tif]

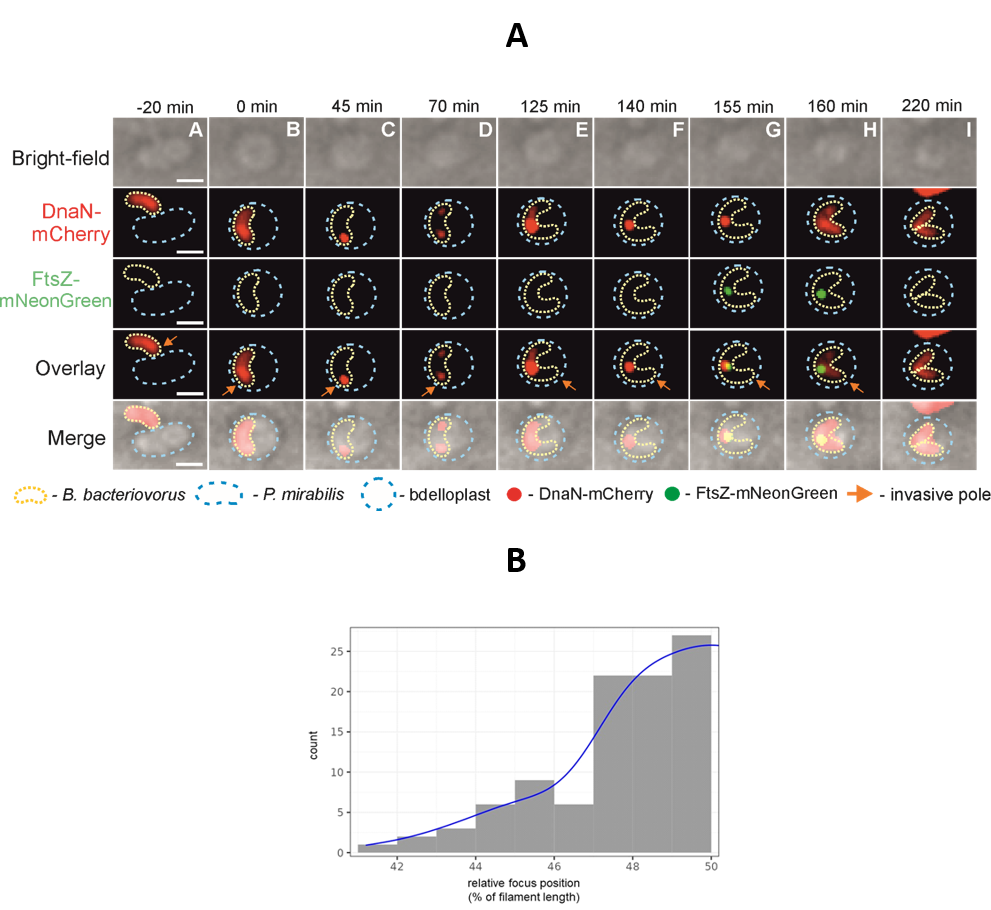

Supplement: FIG S5 [file mbio.00772-23-s0008.tif]
