# Supplementary material for: Binary or Nonbinary Fission? Reproductive Mode of a Predatory Bacterium Depends on Prey Size
Source: mBio. 2023 May 10;14(3):e00772-23. doi: 10.1128/mbio.00772-23 (PMC10294633; doi:10.1128/mbio.00772-23)
Supplement: TABLE S2 [file mbio.00772-23-s0003.docx]

**Table S2. Number of *B. bacteriovorus* progeny cells formed in *E. coli* S17-1 and S. *flexenri* preys that differ in sizes.**

|  | **Number of progeny cells** | | |
| --- | --- | --- | --- |
|  | Prey cell length [µm] | | |
|  | 2.00-2.95 | 3.00-3.99 | 4.00-5.20 |
| *E. coli* S17-1 | 3 (2.5%)*^a^*  4 (46.3%)  **5 (51.2%)** | 4 (2.9%)  **5 (41.2%)**  **6 (38.2%)**  7 (11.8%)  8 (5.9%) | **6 (33.3%)**  **7 (33.3%)**  **8 (22.2%)**  9 (11.2%) |
| *S. flexneri* | 4 (17.4%)  **5 (43.6%)**  6 (21.6%)  7 (17.4%) | 4 (5.1%)  5 (19%)  **6 (50%)**  7 (19%)  8 (6.9%) | **6 (26.3%)**  **7 (31.6%)**  **8 (42.1%)** |

*^a^*in the brackets, percentage of prey cells
