# Supplementary material for: Binary or Nonbinary Fission? Reproductive Mode of a Predatory Bacterium Depends on Prey Size
Source: mBio. 2023 May 10;14(3):e00772-23. doi: 10.1128/mbio.00772-23 (PMC10294633; doi:10.1128/mbio.00772-23)
Supplement: TEXT S1 [file mbio.00772-23-s0001.docx]

**Supplemental Materials**

**Materials and Methods**

**Western blot analysis**

50 mL of overnight culture of *B. bacteriovorus* cells was spun down at 6000 rpm for 20 min at 20°C and resuspended in 5 mL fresh Ca-HEPES buffer. An overnight culture of *E. coli* ML35 cells was spun down at 5000 rpm for 10 min at 20°C, and the cells were washed and back diluted to OD_600_ = 1.0 with Ca-HEPES buffer. The concentrated culture of *B. bacteriovorus* mixed with 4 mL of diluted *E. coli* cells and added 4 ml of Ca-HEPES buffer to final volume 12 mL. After 180 min of incubation, the culture was spun down and resuspended in 300 µL of Ca-HEPES buffer with protease inhibitor cocktail (Pierce Protease Inhibitor Tablets, Thermo Scientific). Cell lysate proteins (10-50 μg in total) were separated in a 10% denaturing polyacrylamide gel before being transferred to a nitrocellulose membrane. The protein was subsequently detected using primary mouse monoclonal anti-mNeonGreen antibody (ChromoTek; 1:1000) and secondary goat anti-mouse IgG- antibody conjugated with horseradish peroxidase (Thermo Scientific;1:5000). Signal from antibody binding was visualized by detecting [chemiluminescence](https://www.sciencedirect.com/topics/biochemistry-genetics-and-molecular-biology/chemoluminescence), which was imaged with Biorad Universal Hood II Gel Doc System.
